# Ganoderic acid loaded nano-lipidic carriers improvise treatment of hepatocellular carcinoma

**DOI:** 10.1080/10717544.2019.1606865

**Published:** 2019-07-30

**Authors:** Mahfoozur Rahman, Shareefa Abdullah Al-Ghamdi, Khalid S Alharbi, Sarwar Beg, Kalicharan Sharma, Firoz Anwar, Fahad A Al-Abbasi, Vikas Kumar

**Affiliations:** a Department of Pharmaceutical Sciences, Shalom Institute of Health & Allied Sciences, Sam Higginbottom University of Agriculture, Technology & Sciences, Allahabad, India;; b Department of Biochemistry, Faculty of Science, King Abdulaziz University, Jeddah, Saudi Arabia;; c Department of Pharmacology, College of Pharmacy, Jouf University, Sakakah 72341, Saudi Arabia;; d School of Pharmaceutical Education and Research, Nanomedicine Research Lab, Jamia Hamdard, New Delhi, India;; e School of Pharmaceutical Education and Research, Jamia Hamdard, New Delhi, India;; f Department of Pharmaceutical Sciences, Faculty of Health Sciences, Natural Product Drug Discovery Laboratory, Sam Higginbottom University of Agriculture, Technology & Sciences, Allahabad, India

**Keywords:** Ganoderic acid, nano-lipid carrier, entrapment efficiency, hepatoprotective, antioxidant, Akt/PKB, HepG2 cell, antioxidants enzymes

## Abstract

This work evaluates nano-lipid carrier of ganoderic acid (GA) and molecular docking on various cancer signaling pathways, an attempt to improve the hepatic condition associated with hepatic carcinoma (HCC) induced by diethyl-nitrosamine (DEN) in Wistar rats. Molecular docking mechanism of GA was performed through binding simulation analysis for various cancer signaling pathway, viz., Bcl-2, Pl3K, NF-κB, Akt/PKB, and Stat-3. Double emulsion solvent displacement method was implied for preparation of GA-loaded nano-lipid carrier. GA-NLCs were evaluated for drug loading capacity, entrapment efficiency, particle size, gastric stability, *in vitro* drug release, cytotoxicity, cellular uptake, and *in vivo* studies including macroscopical, hepatic injury markers, non-hepatic, biochemical, antioxidant parameters, and histopathological evaluation. HCC was induced by intraperitoneal injection of DEN (200 mg/kg). Both *in vivo* and molecular docking results were compatible in establishing the alteration in hepatic nodules, hepatic, non-hepatic, and antioxidant parameters, in a significant manner (*p* < .001) by GA and GA-NLC along with signal alteration of Bcl-2, Pl3K, NF-κB Akt/PKB, and Stat-3 pathway. Histopathological observation confirmed and supported the above result by GA and GA-NLC. On the basis of our results, we can advocate that, GA interferes with various cancer signaling proteins involved in pathogenesis of cancer and was able to cease the progression of disease. Additionally, GA-NLCs proved its chemoprotective effect against the DEN-induced HCC by modulation of hepatic and non-hepatic parameters through various mechanisms.

## 1. Introduction

Hepatocellular carcinoma (HCC) is third most common cause of mortality in the world (Lawrence et al., 1995). It accounts for 70–85% of primary malignancy of liver and its frequent development is associated with chronic inflammation either due to persistent of hepatitis infection or continues state of fatty liver. More than 6,20,000 cases are reported annually with 80% of these cases reported from Africa, South East Asia, and China (Bosch et al., 2004; Perz et al., 2006). Medical and clinical fraternity is aware that effectual available treatment of HCC is limited, only surgical resection and liver transplantation are the viable options, but both of these treatments have limitations either due to condition of the patient or due to late detection of HCC stage (Choi et al., 2009). Diethyl-nitrosamine (DEN) is commonly found in food products, i.e. soybean, cheese, alcoholic beverages, processed meats, cosmetics, agriculture chemicals, and tobacco products. DEN is well known to disrupt the nuclear enzymes which are implicated in DNA replication/repair and its repeated administration can cause HCC (Sadek et al., 2017). As on date several synthetic chemotherapeutic agents/drugs are available, but they are inadequate and have serious adverse effects during treatment regime. Therefore, there is urgent requirement of effective, well tolerated chemotherapeutic agents in the management of liver cancer that can minimize the mortality and morbidity in patients (Sadek et al., 2018).

Tradition medicinal plants including mushroom have been in use from centuries in treatment of liver diseases. *Ganoderma lucidum* mushroom forming white rot fungus is not an exception and has extensively found priority over other plants for liver disorder (Lin & Zhang, 2004; Bishop et al., 2015). Ganoderic acid (GA), a bioactive secondary metabolite of basidiomycetous fungi+*Ganoderma lucidum* is considered as the most important pharmacologically active constituent of *G. lucidum* (Lin & Zhang, 2004; Bishop et al., 2015). Scientific literature is available for its hepato-protective, antihypertensive, hypocholesterolemia, anti-histaminic, antitumor, and antiangiogenic nature. It is known for its effect against free radicals and minimizing cell damage induced by mutagens in animal model along with its immunity enhancing and hepatoprotective properties (Wang et al., 2007; Weng et al., 2009).

The present work is an effort to explore the possible mechanism of action through five signal molecules such as AKT (also known as PKB, protein kinase B), phosphatidyl inositol-4,5-bisphosphate 3-kinase (PI3K), nuclear kappa B (NF-KB), Stat-3, and anti-apoptotic protein B cell lymphoma-2 (Bcl-2) in our model of study (Kimura et al., 2002; Thorgeirsson & Grisham, 2002; Kang & Reynolds, 2009; Fernandez-Ros et al., 2015). All these molecules play a key role in expansion and progression of cancer. The importance of these signal molecules in cancer development made us focus to target these regulatory molecules in anticancer therapy (Pandey et al., 2018). Furthermore, researchers have developed several biomolecules to enhance the bioavailability of such drug molecules into nanoformulation using lipids carrier for their delivery to target cells (Kumar et al., 2017; Pandey et al., 2018). Müller et al. in early 2000 have developed nanostructured lipidic carrier (NLC) for various drugs delivery including chemotherapeutic molecules by use of NLC to the tumor sites. This system of drug delivery was able to accumulate chemotherapeutic agent at tumor site through leaky vasculature, that restricted the growth of these cells (Müller et al., 2002a,b). The major breakthrough was development of NLCs developed by use of biodegradable and biocompatible excipients, i.e. solid and liquid lipids to form an imperfect matrix with a big cavity for easy and better entrapment of drug molecules (Müller et al., 2002a). The present study is based on development of GA encapsulated NLC, referred as GA-NLC. It offers optimistic physiochemical characteristics including better encapsulation efficiency (EE) with particle diameter of 156 nm. The present work on GA-NLC has shown superior cytotoxic activity and cellular uptake toward hepatic cancer cell line (HepG2) supported with histopathological study and determination of other parameters including hepatic injury markers, biochemical parameters, and antioxidants action (Valko et al., 2006; Nair et al., 2007; Ayala et al., 2014; Schieber & Chandel, 2014; Kumar et al., 2017) in HCC of Wistar rats as compared to GA solution.

## 2. Materials and methods

### 2.1. Materials

Ganoderic acid and DEN were purchased from the Sigma Aldrich (St. Louis, MO). Solid lipids, i.e. Cap MCM C10 were obtained from M/s Gattefosse (La Défense Cedex, France). Phospholipid 90G (PL-90G) was received from Lipoid GmBH (Ludwigshafen, Germany). Poloxamers (Kolliphor^®^ P 188, Kolliphor^®^ P 338, and Kolliphor^®^ P 407) were obtained from BASF (Mumbai, India). Tween 80 was purchased by Fisher Scientific Pvt. Ltd. (Mumbai, India) and procured by us. HPLC grade solvents were procured from local vendors. Alkaline phosphatase (ALP) kit, albumin kit, aspartate transaminase (AST) kit, alanine transferase (ALT) kit, and total protein estimation kit were procured from Span Diagnostics (Surat, India).

### 2.2. Molecular docking study

Molecular binding of GA and for their better understanding, we performed molecular docking studies at the RAC-alpha serine/threonine-protein kinase receptor catalytic ligand binding site (PDBID: 3o96), Bcl-2 receptor (PDBID: 2o21), NF-kB receptor (PDBID: 5az5), Pi3kB receptor (PDBID: 5oq4), and tyrosine-protein kinase receptor (PDBID: 4c61). The docking simulations of GA were performed by using, version 9.6 implemented from Schrodinger software suite. The ligand in 3D format with the help of build panel was employed and kept in ready mode for docking studies by ligprep application.

### 2.3. AMDET and MM/GBSA study

Ganoderic acid and co-crystal ligand IQO, 43B, MBL, A3W, and LMM were admitted to ADMET study by QikProp (Qikprop, Version 3.5). It provides wide ranges on properties of the particular drug to compare with other 95% of known drugs further from this partition coefficient, intestinal blood barrier permeability and oral absorption ability, which were also assayed. The free binding energy of complexes of GA and co-crystal ligand IQO, 43B, MBL, A3W, and LMM against 3o96, 2o21, 5az5, 5oq4, and 4c61 respectively was done by using MM/GBSA energy calculations implemented in Prime module of the Schrodinger molecular model package. The trajectory frames to last complex structures from the last 10 ns (i.e. one trajectory frame in each 2 ns) were marked and binding free energies of corresponding structures were determined by VSGB 2.0 solvation model and OPLS3 force field to predict binding free energies.

### 2.4. Preparation of ganoderic acid loaded nanostructured lipid carrier (GA-NLC)

#### 2.4.1. Pseudo ternary phase diagram

The phase diagram was constructed as per the method of (Kumar et al., 2016). The lipid ratio composition of solid lipid (Cap MCM C10), liquid lipid (Capmul PG8) was 3:1. Tween 80 and PL-90G were used as surfactant and co-surfactants mixtures (*S*_mix_) in ethanol in various ratio with distilled water as aqueous phase. Furthermore, the weight amounts of *S*_mix_ and the lipid mixtures were heated at same temperature. Microemulsion (ME) regions was obtained at 60 °C with fixed amount of *S*_mix_ and lipid mixtures (i.e. 10:0–0:10 w/w) with further titration with aqueous phase to obtain a transparent titrated mixture. This titration was continued until turbidity was observed or vice versa. Whereas in the back titration, *S*_mix_ was added in aqueous phase and titrated with lipid mixtures, for rest above procedure was followed. Overall, after obtaining resultant quantities of titrants and aqueous ratio, the weight percent was determined coinciding with boundary points in Gibbs phase triangle (Amarji et al., 2015; Kumar et al., 2016).

#### 2.4.2. Preparation of NLCs

The GA-loaded NLCs were developed by hot ME technique (Heidolph, Silent Crusher M, Heidolph, Germany). In brief, GA and lipid mixtures (Cap MCM C10, Capmul PG8) were melted with addition of ethanol solution of PL-90G and Tween 80 with addition of sufficient water that resulted in our primary ME. This was processed at 60–70 °C with continuous stirring and sonication for 15 s at 3 W by probe sonicator (Sonicator 3000, Misonix). The resultant hot ME was incorporated into 1% w/v poloxamer by micro-syringe under high shear. The shear homogenizer was used at the 9000 rpm for 10 min followed by magnetic stirring (Remi, Mumbai, India) at 600 rpm for 2–3 h. Unentrapped drug was removed from the dispersed NLCs by cellulose dialysis bag (MWCO 10 kDa), by passing double distilled water and dimethyl formamide (DMF) mixture in the ratio of 2:1.

### 2.5. Characterization of NLCs

#### 2.5.1. Size, polydispersity index, zeta potential, and surface morphology

Characterization of drug NLCs formulation in the terms of size, zeta potential, and PDI was determined by Zetasizer (Nano ZS, Malvern, UK). Dynamic light scattering (DLS) method was employed to determine size and PDI of NLC (Kumar et al., 2016), whereas the determination of zeta potential was made on principle of electrophoretic mobility in the presence of electric field. Furthermore, the sample preparation was done by diluting the NLCs with 10–20 times of deionized water, filled in polystyrene cuvettes to analyze the above parameters, whereas transmission electron microscopy (TEM) is employed for determination of surface morphology. A drop of diluted NLCs was added on coated grid surface and stained by a drop of 1% phosphotungstic acid (PTA) on grid surface. Excess fluid was removed from the surface and kept for drying at the room temperature to visualize under microscope (JEM-2100F, M/s Jeol, Tokyo, Japan).

#### 2.5.2. Field emission scanning electron microscopy (FE-SEM) studies of GA-NLC

Generally, standard electron microscope uses high energetic electrons produced by heating a tungsten filament (electron gun) or by a crystal of LaB6, but in case of field emission electron microscope only cold source is use. In our experiment, the electron emission was made from the surface of conductor, which has sharp tungsten needle (tip diameter 10–100 nm) with acceleration voltage at magnitude of 0.5–30 kV under extreme vacuum (∼10–6 Pa) in column of microscope, followed by addition of NLCs drop on Nucleopore Track-Etch membrane and dried at room temperature and coated by sputter coating of platinum. Overall, the images were obtained at –140 °C and a voltage of 15kV using FE-SEM (SU-8010 ultra-high-resolution cold emission, Hitachi, Tokyo, Japan).

#### 2.5.3. Drug loading capacity and encapsulation efficiency

The entrapment efficiency is the percentage of drug which is successfully entrapped and adsorbed into NLCs. The drug loading capacity and EE were measured by application of lysis method. The unentrapped drug containing NLCs suspension was lysed by ethanol. Further, the lysed sample was diluted with mobile phase and filtered out, filtrate was analyzed by LC–MS (Ahmad et al., 2018) for the quantification of EE and drug loading capacity using the following equations:

(1) $$\mathrm{DL}\;(\%)=\frac{\text{total amount of GA encapsulated in NLC}}{\text{total amount of NLC weight}}\times 100$$

(2) $$\mathrm{EE}\;\left(\%\right)=\frac{\text{total amount of GA}-\text{free amount of GA}}{\text{total amount of GA}}\times 100$$

#### *2.5.4.* In vitro *gastrointestinal stability*

Briefly, *in vitro* gastrointestinal stability of NLCs was carried from developed formulations (aliquot 2 mL NLCs suspension). NLCs were added to 250 mL of simulated gastric fluid for 2 h and simulated intestinal fluid for 6 h time duration. One milliliter of the sample was withdrawn for analyses of particle size, PDI, *ζ* potential, and entrapment efficiency.

### 2.6. *In vitro* drug release

*In vitro* studies for NLCs were carried out in dialysis bag, simulated intestinal fluid of pH 6.8 for 24 h at 100 rpm and temperature maintained at 37 ± 0.5 °C was used. In this context, the dialysis membrane was prepared with molecular weight 12 kDa (M/s Himedia Limited, Mumbai, India). NLCs with equivalent weight of 25 mg of the drug were added in dialysis bag containing 1.5 mL of dissolution medium. 0.5 mL of sample was withdrawn at regular interval of time, replenished with equal volume of fresh dissolution medium to maintain the sink conditions. The data obtained were analyzed with in-house software ZOREL (Kumar et al., 2016; Lozoya-Agullo et al., 2018).

### 2.7. Cell culture and cell viability study

Human hepatocellular carcinoma cell line (HepG2) was grown in Dulbecco’s modified Eagle’s medium (DMEM), supplemented with 10% (v/v) fetal bovine serum (FBS), 1 mM l-glutamine, 100 U/mL penicillin, and 100 mg/mL streptomycin. The cells were incubated 37 °C with 5% CO_2_/95% air environment. MTT (3-[4,5-dimethylthiazol-2-yl]-2,5-diphenyltetrazolium bromide) assay was performed for estimation of cytotoxic effect of GA on these cell lines (Jain et al., 2014; Beg et al., 2015; Pandey et al., 2018). Briefly, GA (10–60 μg/mL) was seeded with the cell lines in the 96 well plate for 24 and 48 h at 37 °C in humidified incubator. After incubation, the MTT (10 μL) was prepared in the phosphate buffer saline and introduced in all plates and again incubated at 37 °C for 2 h. Furthermore, the optical density of the said solution was calculated at 570 nm using an ELISA plate reader (BioTek, Winooski, VT) and cell viability was determined.

$$\text{\% cell inhibition}=100-\left\{\frac{\mathrm{test}}{\mathrm{control}}\right\}\times 100$$

Graph pad prism software was used to determine the half maximal inhibitory concentration (IC_50_).

### 2.8. Qualitative cellular uptake experiment

Qualitative cell uptake assay was performed with the coumarin-6 fluorescent co-encapsulated GA-NLC. The preparation of coumarin-6 was initiated by addition of organic phase (DMF) and coumarin-6-co-encapsulated GA-NLC preparation in it by a procedure explained in section 2.4.2 (Jain et al., 2014; Sonawane et al., 2014). Once the cells reached confluency, exhausted media was removed, cells were washed thrice with Hank’s buffered salt solution (HBSS) (PAA Laboratories GmbH, Pasching, Austria). The coumarin-6-GA-NLC containing equivalent to 1 μg/mL free coumarin-6, was added onto cells and incubated for 2 h. Furthermore, the extracellular particles were removed by washing with HBSS (5×), cells were fixed by 3% paraformaldehyde (Merck, Mumbai, India) to make permeable with 0.2% Triton X-100 (Sigma, St. Louis, MO). Cells are observed under CLSM (Olympus FV1000, Tokyo, Japan) and photomicrograph was taken at required magnifications.

### 2.9. Animals

Albino male Wistar strain rats (weighing 160–200 g) were acclimatized under the standard condition (temperature 25 ± 2 °C; 12/12 h light and dark cycle). The rats were fed with the standard rat chow and water *ad libitum*. The complete experimental procedures were reviewed and permitted by Institutional Animal Ethical Committee regulations approved by the Committee for the purpose of Control and Supervision of Experiments on Animals, (Reg. No. SIP/IAEC/PCOL/06/2017.

### 2.10. Induction of HCC

The rats were treated with the single intraperitoneal injection of DEN in phosphate buffer (200 mg/kg) except normal control and normal control treated with GA and after the 10 days, the alpha fetoprotein (AFP) level was estimated for confirmation of HCC development.

### 2.11. Experimental procedure

The rats were grouped into seven, with six animals in each group. Group I control group of animals received a single daily dose of 0.9% w/v normal saline 5 mL/kg/day p.o. (orally). Group II treated with GA (100 mg/kg) 5 mL/kg/day p.o. for 14 weeks, group III DEN control received single dose of DEN (200 mg/kg) in phosphate buffer, group IV DEN control treated with GA (25 mg/kg) single dose 5 mL/kg/day p.o. for 14 weeks, group V DEN control treated with GA (50 mg/kg) single dose 5 mL/kg/day p.o. for 14 weeks, group VI DEN control treated with GA (100 mg/kg) single dose 5 mL/kg/day p.o. for 14 weeks, and group VII DEN control treated with GA-NLC, (25 mg/kg) single dose 5 mL/kg/day p.o. for 14 weeks.

Body weight of all groups was measured at regular interval of time; at the end of experiment, the rats were euthanized by cervical dislocation. Samples (∼0.5 mL blood) were collected from the retro-orbital plexus from time to time in heparinized micro-centrifuge tubes, processed and stored at 4 °C for further use (Verma et al., 2017). Serum was separated by centrifugation at 5000 rpm at 37 °C and process for determination of biochemical and antioxidant parameters. The liver tissues were kept for microscopical and histopathological examination.

### 2.12. Hepatic tissue injury markers

Hepatic tissue injury markers such as AFP, carcinoembryonic antigen (CEA), gamma-glutamyl transferase (GGT), AST, ALP, and alanine transaminase (ALT) (Kumar et al., 2017) were estimated as per the instruction of Kits manufacturer (Span Diagnostic, Surat, India).

### 2.13. Biochemical parameters

Biochemical parameters such as total protein, globulin and albumin were estimated using the method adopted by Kumar et al. with slight modification (Kumar et al., 2017; Pandey et al., 2018).

### 2.14. Antioxidant parameters

An antioxidant parameter glutathione (GSH), myeloperoxidase (MPO), glutathione-S-transferase (GST), glutathione peroxidase (GPx), catalase (CAT), superoxide dismutase (SOD), and malonaldehyde (MDA) were estimated by standard method with minor modification (Kumar et al., 2017; Pandey et al., 2018). Vitamin C and E were estimated using the reported method with minor modification.

### 2.15. Histopathology examination

Liver tissues were prepared from all group rats, fixed in the formalin (10%), dehydrated in gradual alcohol (50–100%), cleared in xylene and were embedded in paraffin. Further, the staining of the tissues was made with the hematoxylin and eosin (H–E) for microscopical observation.

### 2.16. Data analysis

Results are shown as mean ± S.E.M. and one-way analysis of variance (ANOVA) followed by Dunnett’s test was used for the determination of total variation inside set of data. *p*<.05 was considered significant.

## 3. Results and discussion

### 3.1. Ganoderic acid docking and (un)binding simulation study of AT1 target

Molecular docking of GA performed against RAC-alpha serine/threonine-protein kinase receptor revealed a common binding orientation of GA in the catalytic binding pocket of RAC-alpha serine/threonine-protein kinase receptor (PDBID: 3o96). Supplementary Figure 1 illustrates the molecular interactions of ligand surface and active site of protein (S1). Herein, we reported the binding pose of GA and also compare with the standard co-crystal ligand (IQO) which is bonded with RAC-alpha serine/threonine-protein kinase protein. GA binding interacts with backbone of RAC-alpha serine/threonine-protein kinase receptor with arg273, thr82, and lys20 (Supplementary Table 1). GA also makes hydrophobic bond with cys296, lys297, val271, and ile84. Its superimposition with co-crystal (IQO) ligand reveals the same interaction that was present in catalytic domain of RAC-alpha serine/threonine-protein kinase receptor (S1).

### 3.2. Ganoderic acid docking and (un)binding simulation study of BCL-2 target

We performed molecular docking of GA against apoptosis regulator Bcl-2 receptor and the docking studies reveal a common binding orientation of GA in the catalytic binding pocket of apoptosis regulator Bcl-2 receptor (PDBID: 2o21). Herein, we report the binding pose of GA and also compare with the standard co-crystal ligand (43B) which was bonded with apoptosis regulator Bcl-2 protein. Ganoderic acid binding interaction was probed with backbone of apoptosis regulator Bcl-2 receptor with arg143 and ala97. It also made hydrophobic bonding with gly142 and asp100 (Supplementary Table 1). GA superimposition with co-crystal (43B) ligand revealed same interaction as in catalytic domain of apoptosis regulator Bcl-2 receptor (Supplementary Figure 2).

### 3.3. Ganoderic acid docking and (un)binding simulation study of NF-kB target

We performed molecular docking of GA against NF-kB receptor; the results illustrated a common binding orientation of GA in the catalytic binding pocket of NF-kB receptor (PDBID: 5az5). Binding pose of GA and its comparison to standard co-crystal ligand (MBL) which is bonded with NF-kB protein were also studied. GA binding interacts with backbone of NF-kB receptor with phe346, phe405, and tyr353 (Supplementary Table 1). GA superimposition with co-crystal (MBL) ligand revealed same interaction as it was found in catalytic domain of NF-kB receptor (Supplementary Figure 3).

### 3.4. Ganoderic acid docking and (un)binding simulation study of Pi3k otarget

We performed molecular docking of GA against Pi3ka receptor; the results illustrated a common binding orientation of GA in the catalytic binding pocket of Pi3k(Mreceptor (PDBID: 5oq4). Binding pose of GA and its comparison with the standard co-crystal ligand (A3W) which is bonded with Pi3k in catalytere also studied. GA binding interacts with backbone of Pi3k wreceptor with gln893 and asp894. GA also makes hydrophobic bonding with asp841, tyr867, ile963, lys890, ser806, and lys807 (Supplementary Table 1). GA superimposition with co-crystal (A3W) ligand revealed same interaction was found in catalytic domain of Pi3kf receptor (Supplementary Figure 4).

### 3.5. Ganoderic acid docking and (un)binding simulation study of JAK-2 (STAT3) target

We performed molecular docking of GA against tyrosine-protein kinase receptor; a common binding orientation of GA in the catalytic binding pocket of tyrosine-protein kinase receptor (PDBID: 4c61) was found. Herein, we reported the binding pose of GA compared with the standard co-crystal ligand (LMM) bonded with tyrosine-protein kinase protein. GA binding interacts with backbone of tyrosine-protein kinase receptor with ser936, tyr931, and asp994. GA also made hydrophobic bonding with gln356, leu855, leu983, and lys882 (Supplementary Table 1). GA superimposition with co-crystal (LMM) ligand revealed same interaction was found in catalytic domain of tyrosine-protein kinase receptor (Supplementary Figure 5).

### 3.6. AMDET and MM/GBSA study

The ADME properties of the GA and co-crystal ligand IQO, 43B, MBL, A3W, and LMM were analyzed using Qikprop. The results of Qikprop analysis of GA and co-crystal ligand IQO, 43B, MBL, A3W, and LMM are illustrated in Supplementary Table 2. Co-crystal ligand IQO, 43B, MBL, A3W, and LMM compounds were chosen for MM/GBSA study for GA. The results of these studies revealed that its binding free energy for MM/GBSA is 3o96, 2o21, 5az5, 5oq4, and 4c61/–84.83, –41.02, –40.09, –15.37, and –69.21, while the binding free energy of the co-crystal ligand IQO, 43B, MBL, A3W, and LMM is 3o96, 2o21, 5az5, 5oq4, and 4c61/–95.67, –38.52, –42.65, 16.59, and –32.53 (Supplementary Table 2).

### 3.7. Phase diagram

Solid lipid (Cap MCM C10) and liquid lipid (Capmul PG8) were selected as solid lipid mixture in the ratio of 3:1. The probable finding of ME region is identified by pseudo-phase diagram, recognized by titration method in various composition analyses. Whereas the boundary points concluded by using Gibbs phase triangle shows various phase behavior, as changes were made in weight fractions of water, lipids mixtures, and *S*_mix_ (Supplementary Figure 6(a,b)). The axis of triangle indicates one of three binary mixtures as aqueous-surfactant, aqueous-oil, and surfactant oil. Moreover, change in the internal ratio of *S*_mix_ and changes in internal ratio of co-surfactant, i.e. ethanol to phospholipids were done with differences in the quantity of *S*_mix_. ME region (Supplementary Figure 6(a,b)) was enhanced with respect PL, by enhancement of ratio of ethanol (up to 2:1). So, it can be concluded that the ratio of co-surfactants, ethanol and PL was optimized as at the ratio of 2:1 for further works. Furthermore, enhancement in the ratio of surfactant and co-surfactant to the level of 2:1 increased the ME region (Supplementary Figure 6(b)).

### 3.8. Preparation and optimizations of NLCs

Hot ME method was utilized to develop the NLCs and Supplementary scheme 1 reveals the hypothesis and steps involved in it. As a lipid mixture, Cap MCM C10 and Capmul PG8 were used as solid and liquid lipid phase, respectively, whereas ethanol was used as a cosolvent for PL90G, for the formation of primary emulsion to be evaporated during NLCs preparations. Nonsignificant (*p*>.05) differences were observed for particle size and PDI of NLCs obtained at various lipid mixtures and *S*_mix_ concentration_,_ selected from ME region. Moreover, NLCs were prepared at the maximum 11.4% lipid mixture and 13.6% *S*_mix_ was with maximum drug load capacity. The secondary surfactant/stabilizers (in external phase), various grade of poloxamers (i.e. Kolliphor^®^ P 188, Kolliphor^®^ 338, and Kolliphor^®^ P407) were undertaken at the different concentrations from 0.25 to 1% w/v for optimization of GA-loaded NLCs in terms of particle size and entrapment efficiency. But with the Kolliphor^®^ P 188, the prepared NLCs were found to have optimum particle size and EE in all poloxamers undertaken for the study. At the low concentration of surfactant, the NLCs showed larger particle size and low loading and poor entrapment capacity and vice versa. Whereas at increased concentration of surfactant (0.25–0.5% w/v) the NLCs were of higher entrapment with low particle size. However, beyond the 0.5% concentration of surfactant, the particle size and entrapment were observed with down trend. Smaller particle size and maximum loading and entrapment were achieved at 0.5% surfactant concentration in all types; however, Kolliphor^®^ P 188 was of better choice among all types of surfactants. Our results supported the earlier data published by other researchers (Garg et al., 2015; Jain et al., 2015), that is 0.5% w/v of poloxamers is optimum concentration for reduced particle size and better entrapment efficiency of nano-lipid carrier. Sonication time and energy of probe sonicator were also optimized as sonication time and energy for more than 15 s and 3 W, it resulted to produce unstable NLCs along with decreased entrapment; the underlying mechanism may be due to agglomeration and formation of larger particle size (data not shown). Increase in sonication time to 15 seconds at 3 W, have increased entrapment efficiency and lowered the size of NLCs; it may be due to increased solubility of GA in lipids, that makes fine NLCs (Avachat & Parpani, 2015; Khurana et al., 2015). Homogenization is key parameter in development of fine NLCs. Their speed and time have great impact on particle size and entrapment. Homogenizer speed and time were optimized from 5000 to 15,000 rpm for the duration of 10–30 min to get uniform size distribution (data not shown) (Mitri et al., 2011). The particle size of NLCs was obtained as 150–180 nm with drug entrapment efficiency in the range of 75–93%. The lowered particle sizes, high drug payload and entrapment efficiency attributed to higher solubility of drug into the lipids.

### 3.9. Characterization

#### 3.9.1. Size, polydispersity index, zeta potential, entrapment efficiency, drug loading capacity, and surface morphology

The optimized NLCs have particle size of 156 nm, PDI of 0.277 (Supplementary Figure 7(A,B) and Supplementary Table 3), zeta potential of –4.99 ± 1.3 mV (Supplementary Figure 8), entrapment efficiency of 86.3 ± 1.5% and drug loading capacity of 12.2 ± 2.11 (Supplementary Table 3). TEM analysis showed the spherical particles with size of approximately 165 nm (Supplementary Figure 7(C)). Further, FE-SEM studies revealed the uniform surface and spherical shape of said formulation (Supplementary Figure 7(D)). Here, we observed some difference in size obtained by TEM with the DLS. These differences could be due to determination of size of NLC in the solid state by TEM, but in case of zetasizer, it measures the hydrodynamic diameters of the formulation in solution. Thus, overall our results agree with findings of other researchers (Avachat & Parpani, 2015; Jain et al., 2015; Khurana et al., 2015) and validate their results.

#### *3.9.2.* In vitro *gastrointestinal stability*

Supplementary Table 4 illustrates particle size, PDI, *ζ* potential, and entrapment efficiency of the optimized NLCs after treating with different GI fluids at pH of 1.2 and 6.8. After all, non-significant differences (*p*>.05) in the formulation characteristics were observed between the NLCs before and after the treatment, thus confirming adequate stability of the optimized NLCs in the GI pH conditions.

### 3.10. *In vitro* release of GA-NLC

Statistical data for 24 hours study, *in vitro* drug release found that biphasic pattern with an initial burst release was observed in first 4 h followed by sustained drug release profile of maximum 35% drug release (Supplementary Figure 9). This may attribute due to the existence of GA encapsulated into NLC, that follows a slow drug release mechanism through surface erosion mechanism. Thus, literature is also available to support a similar mechanism which governs it for the drug release from GA-NLC (Kumar et al., 2016).

### 3.11. *In vitro* cytotoxic activity

The *in vitro* cytotoxicity was determined by MTT assay. Toxicity linked with GA solution, GA-NLC, and blank NLC formulation following incubation with HepG2 cells for 24 and 48 h was determined (Supplementary Figure 10(A) and 10(B)). The cytotoxicity study is used to report the cell viability by applying different concentration of said formulations, resulted in reduction of cell viability with the concentration dependent manner at specified time for GA and GA-NLCs.

Therefore, the results suggest that GA retained its antitumor efficacy even after encapsulated onto NLC (Ding et al., 2017). Also, GA-loaded NLC proved better anticancer action compared to that seen with GA only. Furthermore, GA-NLC found higher reduction in cell viability over GA solution only.

*GA* (Supplementary Figure 10(A,B)). Moreover, the % cell viability has a coherence with drug sensitivity assay (IC_50_). Whereas, the greater number of living cells was seen at higher IC_50_ in case of GA and least number of viable cells with GA-NLC at lower value of IC_50_ in 48 h of study (Supplementary Figure 10(A,B) and Table 1).

**Table 1.** MTT viability assay of GA solution and optimized GA-NLC.

Formulations	IC_50_ (µg/mL)	
	24 h	48 h
GA solution	48.123	40.012
GA-NLC	40.121	32.612

IC_50_: half maximal inhibitory concentration.

The GA-NLC shows significant (*p<.*05) cytotoxicity compared to GA solution after 24 and 48 h. Data are expressed as mean ± SD (*n* = 6).

### 3.12. Cell uptake study

**Figure 1.**
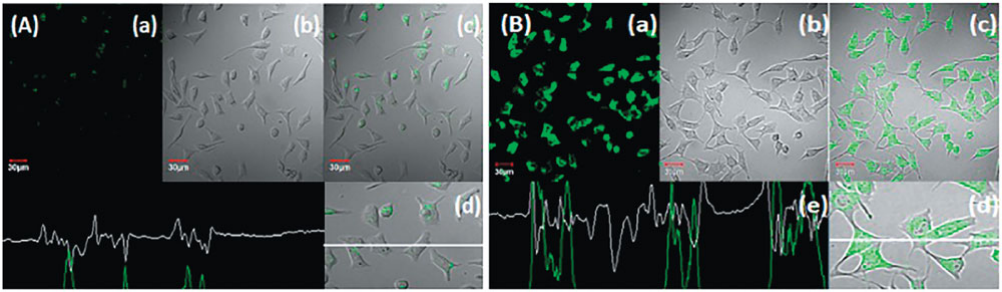
Uptake of (A) free coumarin-6 (C-6) and (B) C-6 loaded GA-nano-lipid carrier, upon incubation at 1 µg/mL for 2 h. In all the images, (a) images under the green fluorescence channel; (b) corresponding differential interface contrast images of HepG2 cells. (c) Superimposition of figure (a) and figure (b). (d, e) In all, the images show horizontal line series analysis of fluorescence along the white line.

The anticancer drugs loaded NLC interacted with hepatic tumor cells are of great importance in HCC targeted therapies (Jain et al., 2014). The qualitative uptake of coumarin-6 and coumarin-6-GA-loaded NLCs in HepG2 cells was evaluated after incubation at 37 °C for 2 h study. The coumarin-6-GA-NLCs show higher and rapid internalization (*p <* .05) in HepG2 cells (Figure 1). Moreover, the confocal images were done by confocal microscopy, which was corroborated by persistent fluorescence signals seen even after 2 h (Figure 1). Therefore, the result suggested better cytotoxicity and greater internalization of GA-NLC over GA, blank NLC, and coumarin-6, respectively (concluded from Supplementary Figure 10(A,B) and Figure 1(A,B)). Whereas GA-NLC formulations exhibited significant (*p <* .05) *in vitro* anti-cancer activity over GA solution; due to possessing of nanosize and lipidic surface of NLCs, these results may attribute to smaller particle size of said formulation with greater uptake as compared to conventional formulation (GA solution), which possesses large particle resulting in lower uptake. Therefore, the present results are supported by earlier studies done by other researchers (Garg et al., 2016).

### 3.13. Effect of GA and GA-NLC on macroscopical character

**Figure 2.**
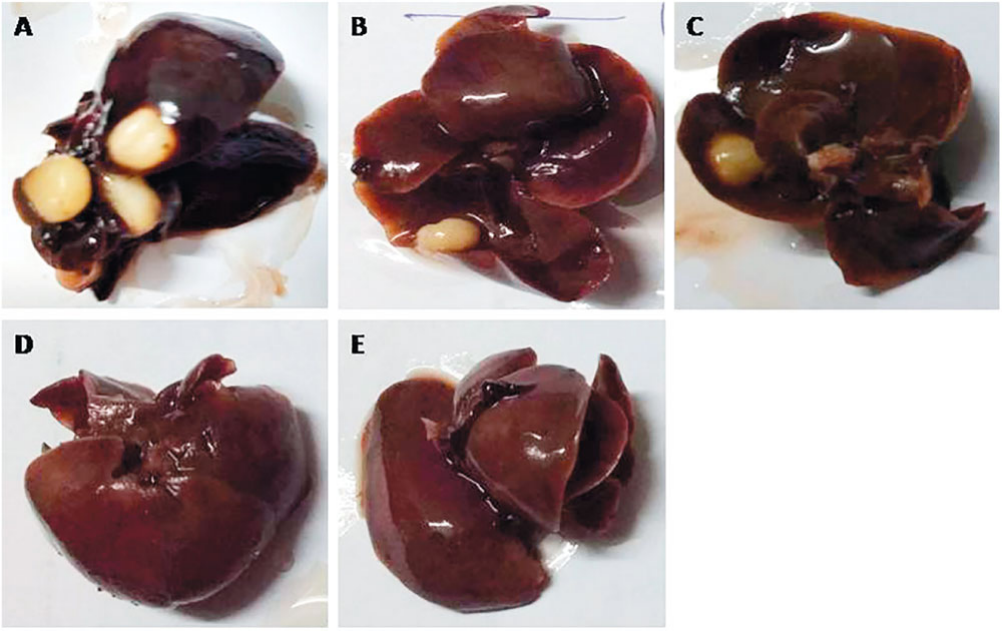
Macroscopical observation of DEN-induced HCC group rats. (A) DEN control group showed the expansion of hepatic nodules (white in color) and decolorization of tissue, (B) DEN control group treated with GA (25 mg/kg) illustrated the expansion of pre-cancerous nodules (white in color) and decolorization of tissue color, which was less as compared to DEN group, (C) DEN control group treated with GA (50 mg/kg) demonstrated the less pre-cancerous nodules (white in color) and decolorization of tissue color, (D) DEN control group treated with GA (100 mg/kg) illustrated the expansion of pre-cancerous nodules and decolorization of tissue color, which was less as compared to other group, and (E) DEN control group treated with GA-NLC (25 mg/kg) illustrated the decolorization of tissue color, which was less as compared to other group rats. *Note*: Normal control and normal control rats treated with GA (100 mg/kg) did not show any sign of precancerous cells and decolorization of skin (data not shown).

Macroscopically, no hepatic nodules or any other abnormalities were observed in the normal control and normal control group rats treated with GA (100 mg/kg). DEN treated rats showed the whitish hepatic nodules distributed on the liver, uneven liver cirrhosis, and surrounding organs like blood vessel were difficult to observe. Whitish hepatic nodules and blood vessel diminished in DEN-induced HCC rats treated with GA (25, 50, and 100 mg/kg) as compared to DEN-induced HCC rats (Figure 2(A–E)). Further, treatment with GA-NLC, rats exhibited absence of formation of nodules in the hepatic tissues and the incidence of hepatic nodules in DEN rats (Table 2 and Supplementary Table 5). The normal control group and normal control treated group do not express any sign of hepatic nodules during the entire course of work, whereas the DEN challenged all groups of rats showed 100% expansion of hepatic nodules. Table 2 and Supplementary Table 5 represent the DEN induced group rats exhibited total 202 number of hepatic nodules with the size of ≤1 mm (101), <3 mm >1 mm (54), and ≥3 mm (47). DEN induced group of rats treated with GA showed the 90.90, 70, and 44.44% of hepatic nodules and the size of ≤1 mm (94, 54, and 22), <3 mm >1 mm (46, 35, and 12), and ≥3 mm (44, 23, and 9) at a dose of 25, 50, and 100 mg/kg, respectively. On the other hand, GA-NLC treated group rats showed the 22.23% of hepatic nodules incidence with a size of ≤1 mm (9), <3 mm >1 mm (7), and ≥3 mm (5).

**Table 2.** The effect of GA-NLC on the number of rats, number of nodules and average number of nodules bearing rats.

S. no.	Groups	Number of rats with nodules/number of rats	Total number of nodules	Relative size (% of number size)		
				≤1 mm	<3 mm >1 mm	≥3 mm
1	DEN control	9/9	202	101 (50)	54 (26.73)	47 (23.26)
2	DEN + GA (25 mg/kg)	10/11	184	94 (51.06)	46 (25)	44 (23.91)
3	DEN + GA (50 mg/kg)	7/10	112	54 (48.21)	35 (31.25)	23 (20.53)
4	DEN + GA (100 mg/kg)	4/9	45	22 (48.88)	14 (31.11)	9 (20)
5	DEN + GA-NLC	2/9	21	9 (42.85)	7 (33.33)	5 (23.80)

Group I and group II did not show any sign of hepatic nodules.

### 3.14. Effect of GA-NLC on animal weight, liver weight, and relative liver weight

Body effect of DEN, GA (25, 50, and 100 mg/kg), and GA-NLC on body weight on the different group of rats is represented in Supplementary Figure 11(a). Increased level of weight exhibited normal control and normal control treated with GA at end of the experimental study. DEN induced group rats showed the increased body weight and similar increased body weight was observed in the GA treated group of rats. The liver weight of the normal control group rats and normal control treated with GA was of almost similar body weight (Supplementary Figure 11(b)). On the other hand, DEN induced group rats showed the increased body weight as compared to the other group rats and dose-dependent treatment of GA reduced the liver weight. GA-NLC treated group rats showed the reduced liver weight almost near to the normal control (Supplementary Figure 11(c)). A similar effect was observed in the relative liver weight. DEN induced group rats showed the increased relative liver weight and dose dependent treatment of GA significantly (*p*<.001) reduced the relative liver weight (Supplementary Figure 11(c)). Furthermore, similar result was observed in the GA-NLC group rats.

### 3.15. Hepatic injury markers and non-hepatic parameters

DEN-induced HCC rats exhibited a significant (*p* < .001) elevation in the serum activities of hepatic specific markers such as AFP, CEA, GGT, AST, ALP, and ALT as compared to normal control, and normal control with GA (Supplementary Figure 12(a–f)).

An up-regulation of AFP content was observed in the DEN group rats as a compared to NC and NC rats received GA (100 mg/kg). GA treatment decreased AFP content as compared to DEN control group rats in a dose-dependent manner. A better reduction of AFP content was observed in the GA-NLC treated group rats (Supplementary Figure 12(a)).

Hepatic injury markers like CEA and GGT were abnormally higher in DEN-induced HCC rats. Dose dependent treatment of GA significantly (*p* < .001) down-regulated the level of these CEA and GGT as compared to DEN-induced HCC rats. On the contrary, GA-NLC significantly (*p* < .001) more downregulated the level of the CEA and GGT as compared to other groups (Supplementary Figure 12(b,c)), which suggested the chemoprotective effect over the hepatic and its functional efficiency.

The variation in serum content of enzymes, enhanced or decreased, reflects the hepatocellular toxicity. Previous literature supports that the expansion of HCC is connected to the elevation of these enzyme markers. Therefore, the estimation of these parameters is important feature to scrutinize early detection of disease (Hietala et al., 2006; Hann et al., 2012; Liu et al., 2014). Human tissues have sufficient quantity of transaminase enzymes such as AST and ALT, during the normal process, these enzymes catalyze shift of amino group from the amino acid and 2-oxoacids, both these enzymes are mostly present in the cytoplasm of cells in muscle, heart, and liver (Kim et al., 2008; Jayakumar et al., 2012). During hepatic toxicity, increase in serum content of ALT and AST is not surprising, in pathological condition, the content of these enzymes increases more than several times as compared to normal level. Various clinical studies suggest that during the hepatic damage, jaundice, and hepatitis, elevation of these enzymes is more than 20–40 times. During the DEN-induced HCC, plasma membrane starts the secretion of cellular cytosolic content to the external milieu. DEN-induced animals showed the marked up-regulation of AST and ALT content assign of cellular injury; this results in increased level of these enzymes in blood after rapture the plasma membrane (Renugadevi & Prabu, 2010). ALP is considered as the important enzyme of the plasma membrane, any alteration in this enzyme demonstrates the alteration of the integrity of plasma membrane. The upregulation of the specific activity of ALP in the serum is a significant indicator to know the loss of integrity in the liver plasma membrane. During DEN metabolism, lipid peroxidation of polyunsaturated fatty acids (PUFAs) via continuous generation of free radicals or oxidative stress is a common feature due to cell damage (Lemaire et al., 1991). GA significantly (*p* < .001) down-regulated the serum ALP, AST, and ALT content at dose-dependent manner (Supplementary Figure 12(d–f)) (Lemaire et al., 1991). The same result was observed by GA-NLC, which reduced the increased level of these enzymes via protecting the membrane integrity or scavenging the free radicals. The results suggest that GA prevents the liver damage by balance between the integrity of plasma membrane and reduction in enzymes leakage from membranes to show signs of hepatic protection.

A similar trend was observed in non-hepatic parameters, i.e. total protein, albumin, globulin, and A/G in the DEN-induced HCC rats. GA has significant (*p* < .001) alteration in content of total protein, albumin, and globulin in a dose-dependent manner. A reduction level of A/G was observed in the DEN group rats, which was significantly (*p* < .001) altered by the GA treatment in a dose-dependent pattern, whereas GA-NLC treatment enhanced the total protein, albumin, globulin, and A/G level almost near the normal control group rats (Supplementary Figure 13(a–d)).

### 3.16. Antioxidant marker

Elevation of antioxidant marker, viz., GSH, MPO, GST, and GPx was observed in DEN treated rats in a dose-dependent manner; treatment of GA has significant (*p* < .001) down-regulation on level of these markers. A similar trend was observed in the GA-NLC treated group rats, where a significant (*p* < .001) reduction was noted in antioxidant marker (Supplementary Figure 14(a–d)).

Other antioxidant parameters including P. Carbonyl, CAT, SOD, and MDA were down-regulated in the DEN group rats and GA significantly (*p* < .001) up-regulated these antioxidant parameters. GA-NLC treatment significantly (*p* < .001) increased them (Supplementary Figure 15(a–d)).

There was significant (*p* < .001) decrease in non-enzymatic antioxidant, viz., vitamin C and E in DEN-treated group and dose-dependent treatment of GA showed marked elevation in level of these non-enzymatic antioxidant parameters (Supplementary Figure 16(a,b)).

Antioxidants play a diverse role in the various biological activities, viz., reduction of prostaglandin synthesis, induction of drug metabolizing enzymes, reversion of carcinogenic effect and scavenging of free radicals are few of them (Iqbal et al., 2003). They also play a significant role inhibition of ROS toxicity by thwarting the ROS formation. Many researchers suggest that natural antioxidants are able to down-regulate the ROS formation and decrease the intracellular oxidative stress. DEN treatment shows the increased level of oxidative stress attributed to overproduction of free radicals, during HCC. DEN-induced HCC, enhanced the level of lipid peroxidation products, viz., malondialdehydes, conjugated dienes, and lipid peroxidation, which suggests the increased oxidative stress on the cellular lipids. Protein carbonyl content is considered as the important marker for the estimation of protein oxidation (Valko et al., 2006; Schieber & Chandel, 2014). Various disease, viz., hepatic toxicity, arthritis, diabetes, Alzheimer’s and inflammatory bowel diseases have been associated with the accumulation of protein. DEN-induced rats showed the significant (*p* < .001) elevation of protein carbonyl due to oxidation of microsomal protein. DEN-induced rats treated with GA demonstrated the significant (*p* < .001) reduction of protein carbonyl content in a dose-dependent manner. GA-NLC treated rats exhibited the down-regulation of protein carbonyl level via averting the oxidation of microsomal protein. Various toxicities and pathological conditions are directly linked to the lipid peroxidation. Therefore, accurate estimation of lipid peroxidation could determine the redox state of cells. The results of DEN-induced HCC group predict the increased level of conjugated dienes and MDA (an indicator and mutagenic product of LPO) a significant sign of imbalance in redox state of cells and could start the oxidative stress (Valko et al., 2006; Schieber & Chandel, 2014). GA-NLC significantly (*p* < .001) decreased the content of MDA and conjugated dienes and preventing the lipid peroxidation. This could be due to the powerful antioxidant character and effect of GA. The CAT and SOD are the first line Defense endogenous antioxidant enzymes against the oxidative damage mediated via superoxide radicals. Primary function of SOD is to reduce the superoxide to hydrogen peroxide radical and water (Prasad et al., 2007). GPx, an armor in liver enzymes initiates the conversion of H_2_O_2_ to nontoxic compounds, glutathione and its associated products upon metabolism act as first line of defense in cells, in condition generated due to oxidative stress and tries to maintain the cell in the reduced state of stability. Detoxification of ROS is governed by GSH whether it is related to cellular activity or to exogeneous factors with ROS. DEN, a carcinogen is well known to disturb all the normal activity of cells by generating ROS that can interact with nucleophilic pool of cells (Khan et al., 2015). Previous studies suggest that the antioxidant enzymes, viz., CAT, SOD, and GSH are present in tumor cells and the same is resembled in our experimental study, suggesting the potential role of DEN in induction of carcinogenic effect. The result suggests the GA scavenges excessive of endogenous free radicals in the body and obstruct the carcinogenesis process.

GST plays an important role in solubilizing the xenobiotics by conjugating the thiol group; this solubility is elevated in hydrophobic substances and plays an important role in storage and elimination of xenobiotics. The GSH conjugate with xenobiotics is then either converted or eliminated to mercapturic acid. Researchers have used this phenomenon to xenobiotics detoxifying enzymes as a therapy to alter the carcinogen effect that involves an activation of xenobiotic and metabolize the toxic products into the nontoxic product (Verma et al., 2018). DEN-induced group showed the up-regulation of GST that may be attributed to decrease coupling of electrophilic intermediate formed during the processes of carcinogenesis. GA alters the GST level and induces more coupling effect to GST required to eliminate the xenobiotics responsible for progression or promotion of cancer in hepatic tissues.

Several studies showed that the excessive generation of oxidative stress and liver damage reduces the level of a non-enzymatic antioxidant (vitamin C and vitamin E). Hydroxyl radicals play a role in the inactivation of enzymatic antioxidants and existence of non-enzymatic antioxidant is presumably necessary to clear these radicals. Non-enzymatic antioxidants, viz., vitamin C and E, act synergistically to scavenge the hydroxyl and other free radicals present in the biological system (Verma et al., 2018). Vitamin C is a water-soluble non-enzymatic antioxidant that eliminates free radicals from the cytosol via acting directly or indirectly. Researchers have proved that the ascorbate molecule may be involved in the reduction of host resistance against cancer. It protects lipoprotein particles and cell membrane injury from oxidative damage via regeneration of antioxidant. Vitamin E acts as the lipid soluble scavenger that averts the lipid peroxidation via initiating the chain reactions of the lipid membrane. It also donates the hydrogen atom from phenolic hydroxyl group to stabilize lipid peroxyl and alkoxyl radical intermediates of LPO. It acts as potent antioxidant due to its capability to infiltrate into the specific site of the membrane, that attribute to protect the cell from highly reactive radicals. DEN-induced group showed the down-regulation of vitamin C and E due to excess generation of free radicals and most utilization of these vitamins to scavenge the radicals during the DEN metabolism. GA treatment showed the upregulation of non-enzymatic antioxidant.

### 3.17. Effect of GA-NLC on histopathology

**Figure 3.**
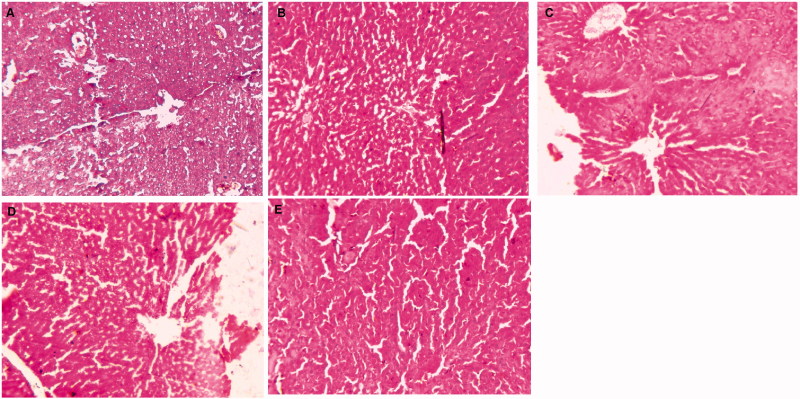
The histopathological evaluation of DEN-induced HCC group rats. (A) DEN control group showed the expansion of hepatic nodules (white in color) and decolorization of tissue, (B) DEN control group treated with GA (25 mg/kg) illustrated the expansion of pre-cancerous nodules (white in color) and decolorization of tissue color, which was less as compared to DEN group, (C) DEN control group treated with GA (50 mg/kg) demonstrated the less pre-cancerous nodules (white in color) and decolorization of tissue color, (D) DEN control group treated with GA (100 mg/kg) illustrated the expansion of pre-cancerous nodules and decolorization of tissue color, which was less as compared to other group, and (E) DEN control group treated with GA-NLC (25 mg/kg) illustrated the decolorization of tissue color, which was less as compared to other group rats. *Note*: Normal control and normal control rats treated with GA (100 mg/kg) did not show any sign of alteration in the histopathology (data not shown).

Normal control group rat’s histopathology demonstrated the normal articulature, small uniform nuclei, typical structure, average central vein, average size of polyhedral shape hepatocytes and small uniform nuclei of cytoplasm. Normal control group rats showed the similar histopathology features as presented in the normal control. DEN induced group rats showed the inflammatory cells, necrosis cells with inflammatory blood vessels, irregular and dark cytoplasm, trabeculae (hepatic parenchyma with thick cords), pseudo acini, and hyperchromatic nuclei. It also showed the hepatic portion with development of proliferation in HSCs, binucleate, masses of eosinophils in vacuolation, irregular macro lipid droplets and irregular structure of cytoplasm. Ganoderic acid treated group rats showed the improvement of histopathological changes such as less altered hepatocytes, inflammatory cells, hepatocellular architecture and absence of proliferation in HSCs portal area and less accumulation of micro-droplets as dose-dependent manner (Table 3). GA-NLC treated group rats showed the improvement of hepatic histopathology in terms of less necrosis in blood cells, inflammatory cells with micro-droplets and also have average size mononucleus and compact cytoplasm (Figure 3(A–E)).

**Table 3.** The effect of GA-NLC on the histopathology character.

S. no.	Histopathology changes	Groups					
		NC	NC + GA (50 mg/kg)	DEN	DEN + GA (25 mg/kg)	DEN + GA (100 mg/kg)	DEN + GA-NLC
1	Necrosis	–	–	+	+	+	+
2	Hydropic degeneration	–	–	+	+	+	–
3	HSCs focal proliferation	–	–	+	+	+	+
4	Bile cysts	–	–	+	–	–	–
5	Pseudo-nucleoli	–	–	+	+	+	–
6	Peliosis hepatis	–	–	+	+	–	–
7	Disorganized hepatic parenchyma	–	–	+	–	–	–
8	Apoptosis	–	–	+	+	–	–
9	Hepatocellular adenoma	–	–	+	+	+	+
10	Cell necrosis	–	–	+	+	–	–
11	Altered basophilic	–	–	+	+	+	+
12	Small dark cytoplasm	–	–	+	–	–	–
13	Enlargement of karyomegaly	–	–	+	+	–	–
14	Macro lipid droplets	–	–	+	+	+	+
15	Diffuse dysplasia	–	–	+	+	–	–
16	Hyperplastic foci	–	–	+	+	–	–

## 4. Conclusions

From the above experimental discussion, it is concluded that GA is able to interact at various cancer signaling proteins, which play significant role in the pathogenesis of cancer. The GA-NLCs are with the particle size of 156 nm with EE >85%, with initial burst release as *in vitro* in duration of 4 h and sustained release for the next 24 h. Further the cytotoxicity of GA-NLCs and cellular uptake against hepatocellular carcinoma cell line HepG2 was superior to GA solution and 6-coumarin solution, respectively. Further, the GA-NLCs also showed better tolerant and antitumor efficacy against hepatic carcinoma. Moreover, the histopathology demonstrated the less alteration in the hepatic tissue via less inflamed blood vessels, less necrosis and less depositions of microdroplets. Therefore, these results implied that the GA-NLCs could enhance its antitumor effect *in vivo* by making balances of hepatic injury markers, biochemicals, and antioxidants markers.

## Supplementary Material

Suppl_Fig_caption.docx

S-Fig_16.jpg

S-Fig_15.jpg

S-Fig_14.jpg

S-Fig_13.jpg

S-Fig_12.jpg

S-Fig_11.jpg

S-Fig_10A_and_10B.jpg

S-Fig_9.jpg

S_Fig-8.pdf

S-Fig_7A-D.jpg

S-Fig_6A_and_6B.jpg

S5.tif

S4.tif

S3.tif

S2.tif

S1.tif

Graphical_Abst.jpg

S-Scheme_1_corrected.jpg

Supplementary_Tables.docx

## References

[CIT0001] Ahmad N, Alam MA, Ahmad R, et al. (2018). Improvement of oral efficacy of Irinotecan through biodegradable polymeric nanoparticles through in vitro and in vivo investigations. J Microencapsul 35:327–43. 29873288 10.1080/02652048.2018.1485755

[CIT0002] Amarji B, Garg NK, Singh SB, et al. (2016). Microemulsions mediated effective delivery of methotrexate hydrogel: more than a tour de force in psoriasis therapeutics. J Drug Target 24(2): 1–14. 10.3109/1061186X.2015.1058804 26204326

[CIT0003] Avachat AM, Parpani SS (2015). Formulation and development of bicontinuous nanostructured liquid crystalline particles of efavirenz. Colloids Surf B Biointerfaces 126:87–97. 25543986 10.1016/j.colsurfb.2014.12.014

[CIT0004] Ayala A, Muñoz MF, Argüelles S (2014). Lipid peroxidation: production, metabolism, and signaling mechanisms of malondialdehyde and 4-hydroxy-2-nonenal. Oxid Med Cell Longev 2014:360438. 24999379 10.1155/2014/360438 PMC4066722

[CIT0005] Beg S, Sharma G, Thanki K, et al. (2015). Positively charged self-nanoemulsifying oily formulations of olmesartan medoxomil: systematic development, in vitro, ex vivo and in vivo evaluation. Int J Pharm 493:466–82. 26211900 10.1016/j.ijpharm.2015.07.048

[CIT0006] Bishop KS, Kao CH, Xu Y, et al. (2015). From 2000 years of *Ganoderma lucidum* to recent developments in nutraceuticals. Phytochemistry 114:56–65. 25794896 10.1016/j.phytochem.2015.02.015

[CIT0007] Bosch FX, Ribes J, Díaz M, et al. (2004). Primary liver cancer: worldwide incidence and trends. Gastroenterology 127:S5–S16. 15508102 10.1053/j.gastro.2004.09.011

[CIT0008] Choi HY, Chong SA, Nam MJ (2009). Resveratrol induces apoptosis in human SK-HEP-1 hepatic cancer cells. Cancer Genomics Proteomics 6:263–8. 19996131

[CIT0009] Ding X, Xu X, Zhao Y, et al. (2017). Tumor targeted nanostructured lipid carrier co-delivering paclitaxel and indocyanine green for laser triggered synergetic therapy of cancer. RSC Adv 7:35086–95.

[CIT0010] Fernandez-Ros N, Iñarrairaegui M, Paramo JA, et al. (2015). Radioembolization of hepatocellular carcinoma activates liver regeneration, induces inflammation and endothelial stress and activates coagulation. Liver Int 35:1590–6. 24836705 10.1111/liv.12592

[CIT0011] Garg NK, Singh B, Kushwah V, et al. (2016). The ligand (s) anchored lipobrid nanoconstruct mediated delivery of methotrexate: an effective approach in breast cancer therapeutics. Nanomedicine 12:2043–60. 27234306 10.1016/j.nano.2016.05.008

[CIT0012] Garg NK, Singh B, Sharma G, et al. (2015). Development and characterization of single step self-assembled lipid polymer hybrid nanoparticles for effective delivery of methotrexate. RSC Adv 5:62989–99.

[CIT0013] Hann HW, Wan S, Myers RE, et al. (2012). Comprehensive analysis of common serum liver enzymes as prospective predictors of hepatocellular carcinoma in HBV patients. PLoS One 7:e47687. 23112834 10.1371/journal.pone.0047687 PMC3480412

[CIT0014] Hietala J, Koivisto H, Anttila P, et al. (2006). Comparison of the combined marker GGT-CDT and the conventional laboratory markers of alcohol abuse in heavy drinkers, moderate dinkers and abstainers. Alcohol Alcohol 41:528–33. 16799164 10.1093/alcalc/agl050

[CIT0015] Iqbal M, Sharma SD, Okazaki Y, et al. (2003). Dietary supplementation of curcumin enhances antioxidant and phase II metabolizing enzymes in ddY male mice: possible role in protection against chemical carcinogenesis and toxicity. Pharmacol Toxicol 92:33–8. 12710595 10.1034/j.1600-0773.2003.920106.x

[CIT0016] Jain A, Jain A, Garg NK, et al. (2015). Surface engineered polymeric nanocarriers mediate the delivery of transferrin–methotrexate conjugates for an improved understanding of brain cancer. Acta Biomater 24:140–51. 26116986 10.1016/j.actbio.2015.06.027

[CIT0017] Jain AK, Thanki K, Jain S (2014). Novel self-nanoemulsifying formulation of quercetin: implications of pro-oxidant activity on the anticancer efficacy. Nanomedicine 10:959–69. 24407148 10.1016/j.nano.2013.12.010

[CIT0018] Jain AK, Thanki K, Jain S (2014). Solidified self-nanoemulsifying formulation for oral delivery of combinatorial therapeutic regimen: part II in vivo pharmacokinetics, antitumor efficacy and hepatotoxicity. Pharm Res 31:946–58. 24135934 10.1007/s11095-013-1214-1

[CIT0019] Jayakumar S, Madankumar A, Asokkumar S, et al. (2012). Potential preventive effect of carvacrol against diethylnitrosamine-induced hepatocellular carcinoma in rats. Mol Cell Biochem 360:51–60. 21879312 10.1007/s11010-011-1043-7

[CIT0020] Kang MH, Reynolds CP (2009). Bcl-2 inhibitors: targeting mitochondrial apoptotic pathways in cancer therapy. Clin Cancer Res 15:1126–32. 19228717 10.1158/1078-0432.CCR-08-0144 PMC3182268

[CIT0021] Khan R, Kazmi I, Afzal M, et al. (2015). Fixed dose combination therapy loperamide and niacin ameliorates diethylnitrosamine-induced liver carcinogenesis in albino Wistar rats. RSC Adv 5:67996–8002.

[CIT0022] Khurana S, Jain NK, Bedi PM (2015). Nanostructured lipid carriers based nanogel for meloxicam delivery: mechanistic, in-vivo and stability evaluation. Drug Dev Ind Pharm 41:1368–75. 25151872 10.3109/03639045.2014.950586

[CIT0023] Kim WR, Flamm SL, Di Bisceglie AM, et al. (2008). Serum activity of alanine aminotransferase (ALT) as an indicator of health and disease. Hepatology 47:1363–70. 18366115 10.1002/hep.22109

[CIT0024] Kimura Y, Taniguchi M, Baba K (2002). Antitumor and antimetastatic effects on liver of triterpenoid fractions of *Ganoderma lucidum*: mechanism of action and isolation of an active substance. Anticancer Res 22:3309–18. 12530080

[CIT0025] Kumar A, Ahuja A, Ali J, et al. (2016). Curcumin-loaded lipid nanocarrier for improving bioavailability, stability and cytotoxicity against malignant glioma cells. Drug Deliv 23:214–29. 24825490 10.3109/10717544.2014.909906

[CIT0026] Kumar V, Bhatt PC, Rahman M, et al. (2017). Fabrication, optimization, and characterization of umbelliferone β-d-galactopyranoside-loaded PLGA nanoparticles in treatment of hepatocellular carcinoma: in vitro and in vivo studies. Int J Nanomedicine 12:6747–58. 28932118 10.2147/IJN.S136629 PMC5600267

[CIT0027] Kumar V, Bhatt PC, Rahman M, et al. (2017). Umbelliferon-α-d-glucopyranosyl-(2_I_→1_II_)-α-d-glucopyranoside ameliorates diethyl-nitrosamine induced precancerous lesion development in liver via regulation of inflammation, hyperproliferation and antioxidant at pre-clinical stage. Biomed Pharmacother 94:834–42. 28802237 10.1016/j.biopha.2017.07.047

[CIT0028] Lawrence TS, Robertson JM, Anscher MS, et al. (1995). Hepatic toxicity resulting from cancer treatment. Int J Radiat Oncol Biol Phys 1:1237–48. 10.1016/0360-3016(94)00418-K 7713785

[CIT0029] Lemaire P, Drai P, Mathieu A, et al. (1991). Changes with different diets in plasma enzymes (GOT, GPT, LDH, ALP) and plasma lipids (cholesterol, triglycerides) of sea-bass (*Dicentrarchus labrax*). Aquaculture 93:63–75.

[CIT0030] Lin Z, Zhang H (2004). Anti-tumor and immunoregulatory activities of *Ganoderma lucidum* and its possible mechanisms. Acta Pharmacol Sin 25:1387–95. 15525457

[CIT0031] Liu X, Hamnvik OP, Chamberland JP, et al. (2014). Circulating alanine transaminase (ALT) and γ-glutamyl transferase (GGT), but not fetuin-A, are associated with metabolic risk factors, at baseline and at two-year follow-up: the prospective Cyprus Metabolism Study. Metabolism 63:773–82. 24726813 10.1016/j.metabol.2014.03.008 PMC4104665

[CIT0032] Lozoya-Agullo I, Araujo F, Gonzalez-Alvarez I, et al. (2018). PLGA nanoparticles are effective to control the colonic release and absorption on ibuprofen. Eur J Pharm Sci 115:119–25. 29248559 10.1016/j.ejps.2017.12.009

[CIT0033] Mitri K, Shegokar R, Gohla S, et al. (2011). Lipid nanocarriers for dermal delivery of lutein: preparation, characterization, stability and performance. Int J Pharm 414:267–75. 21596122 10.1016/j.ijpharm.2011.05.008

[CIT0034] Müller RH, Radtke M, Wissing SA (2002a). Nanostructured lipid matrices for improved microencapsulation of drugs. Int J Pharm 242:121–8. 12176234 10.1016/s0378-5173(02)00180-1

[CIT0035] Müller RH, Radtke M, Wissing SA (2002b). Solid lipid nanoparticles (SLN) and nanostructured lipid carriers (NLC) in cosmetic and dermatological preparations. Adv Drug Deliv Rev 54:S131–S55. 12460720 10.1016/s0169-409x(02)00118-7

[CIT0036] Nair U, Bartsch H, Nair J (2007). Lipid peroxidation-induced DNA damage in cancer-prone inflammatory diseases: a review of published adduct types and levels in humans. Free Radic Biol Med 43:1109–20. 17854706 10.1016/j.freeradbiomed.2007.07.012

[CIT0037] Pandey P, Rahman M, Bhatt PC, et al. (2018). Implication of nano-antioxidant therapy for treatment of hepatocellular carcinoma using PLGA nanoparticles of rutin. Nanomedicine (Lond) 13:849–70. 29565220 10.2217/nnm-2017-0306

[CIT0038] Perz JF, Armstrong GL, Farrington LA, et al. (2006). The contributions of hepatitis B virus and hepatitis C virus infections to cirrhosis and primary liver cancer worldwide. J Hepatol 45:529–38. 16879891 10.1016/j.jhep.2006.05.013

[CIT0039] Prasad L, Khan TH, Jahangir T, et al. (2007). Abrogation of DEN/Fe-NTA induced carcinogenic response, oxidative damage and subsequent cell proliferation response by *Terminalia chebula* in kidney of Wistar rats. Pharmazie 62:790–7. 18236787

[CIT0040] Renugadevi J, Prabu SM (2010). Cadmium-induced hepatotoxicity in rats and the protective effect of naringenin. Exp Toxicol Pathol 62:171–81. 19409769 10.1016/j.etp.2009.03.010

[CIT0041] Sadek KM, Abouzed TK, Abouelkhair R, Nasr S (2017). The chemo-prophylactic efficacy of an ethanol *Moringa oleifera* leaf extract against hepatocellular carcinoma in rats. Pharm Biol 55:1458–66. 28345375 10.1080/13880209.2017.1306713 PMC6130573

[CIT0042] Sadek KM, Lebda MA, Nasr NE, et al. (2018). Role of lncRNAs as prognostic markers of hepatic cancer and potential therapeutic targeting by S-adenosylmethionine via inhibiting PI3K/Akt signaling pathways. Environ Sci Pollut Res Int 25:20057–70. 29748795 10.1007/s11356-018-2179-8

[CIT0043] Schieber M, Chandel NS (2014). ROS function in redox signaling and oxidative stress. Curr Biol 24:R453–62. 24845678 10.1016/j.cub.2014.03.034 PMC4055301

[CIT0044] Sonawane R, Harde H, Katariya M, et al. (2014). Solid lipid nanoparticles-loaded topical gel containing combination drugs: an approach to offset psoriasis. Expert Opin Drug Deliv 11:1833–47. 25078031 10.1517/17425247.2014.938634

[CIT0045] Thorgeirsson SS, Grisham JW (2002). Molecular pathogenesis of human hepatocellular carcinoma. Nat Genet 31:339–46. 12149612 10.1038/ng0802-339

[CIT0046] Valko M, Rhodes CJ, Moncol J, et al. (2006). Free radicals, metals and antioxidants in oxidative stress-induced cancer. Chem-Biol Interact 160:1–40. 16430879 10.1016/j.cbi.2005.12.009

[CIT0047] Verma A, Ahmed B, Anwar F, et al. (2017). Novel glycoside from *Wedelia calendulacea* inhibits diethylnitrosamine-induced renal cancer via downregulating the COX-2 and PEG2 through nuclear factor-κB pathway. Inflammopharmacology 25:159–75. 28155120 10.1007/s10787-017-0310-y

[CIT0048] Verma A, Singh D, Anwar F, et al. (2018). Triterpenoids principle of *Wedelia calendulacea* attenuated diethynitrosamine-induced hepatocellular carcinoma via down-regulating oxidative stress, inflammation and pathology via NF-kB pathway. Inflammopharmacology 26:133–46. 28608141 10.1007/s10787-017-0350-3

[CIT0049] Wang G, Zhao J, Liu JW, et al. (2007). Enhancement of IL-2 and IFN-gamma expression and NK cells activity involved in the antitumor effect of ganoderic acid Me in vivo. Int Immunopharmacol 7:864–70. 17466920 10.1016/j.intimp.2007.02.006

[CIT0050] Weng CJ, Chau CF, Yen GC, et al. (2009). Inhibitory effects of *Ganoderma lucidum* on tumorigenesis and metastasis of human hepatoma cells in cells and animal models. J Agric Food Chem 57:5049–57. 19422227 10.1021/jf900828k

