# Supplementary material for: Ganoderic acid loaded nano-lipidic carriers improvise treatment of hepatocellular carcinoma
Source: Drug Deliv. 2019 Jul 30;26(1):782–93. doi: 10.1080/10717544.2019.1606865 (PMC6711158; doi:10.1080/10717544.2019.1606865)
Supplement: Suppl_Fig_caption.docx [file IDRD_A_1606865_SM2297.docx]

**Supplementary Figure Caption**

**S-Scheme 1.** The formulation, characterization, *in vitro* and *in vivo* studies of GA encapsulated NLCs.

**S-Graphical Abstract:** Graphical Abstract represents: I) GA-NLC II) Characterizations III) In vitro Drug release IV) In vitro cytotoxicity V) Cell Uptake VI) Liver Cancer in rat and their treatment VII) Histopathological images.

**S-Fig 1:** showed the molecular docking study of ganoderic acid (GA) on RAC-alpha serine/threonine-protein kinase receptor. **(a)** The binding mode of GA (brown) is shown in the RAC-alpha serine/threonine-protein kinase receptor active site and important residues are highlighted with cyan stick, **(b)** Ligplot of GA is shown in the RAC-alpha serine/threonine-protein kinase receptor active site, **(c)** The binding mode of Co-crystal ligand (IQO)(brown) is shown in the RAC-alpha serine/threonine-protein kinase receptor active site and important residues are highlighted with cyan stick, **(d)** Ligplot of Co-crystal ligand (IQO) is shown in the RAC-alpha serine/threonine-protein kinase receptor active site and **(e)** Superimposition of GA (cyan) at catalytic domain of RAC-alpha serine/threonine-protein kinase receptor with Co-crystal ligand (IQO) (green).

**S-Fig 2:** showed the molecular docking study of GA on RAC-alpha serine/threonine-protein kinase receptor. **(a)** The binding mode of GA (brown) is shown in the Apoptosis regulator Bcl-2 receptor active site and important residues are highlighted with cyan stick, **(b)** Ligplot of GA is shown in the Apoptosis regulator Bcl-2 receptor active site, **(c)** The binding mode of Co-crystal ligand (43B)(brown) is shown in the Apoptosis regulator Bcl-2 receptor active site and important residues are highlighted with cyan stick, **(d)** Ligplot of Co-crystal ligand (43B) is shown in the Apoptosis regulator Bcl-2receptor active site and **(e)** Superimposition of GA (cyan) at catalytic domain of Apoptosis regulator Bcl-2 receptor with Co-crystal ligand (43B) (green).

**S-Fig 3:** showed the molecular docking study of GA on NF-kB receptor. **(a)** The binding mode of GA (brown) is shown in the NF-kB receptor active site and important residues are highlighted with cyan stick, **(b)** Ligplot of GA is shown in the NF-kB receptor active site, **(c)** Ligplot of Co-crystal ligand (MBL) is shown in the NF-kB receptor active site, **(d)** Ligplot of Co-crystal ligand (43B) is shown in the Apoptosis regulator Bcl-2receptor active site and **(e)** Superimposition of GA (cyan) at catalytic domain of NF-kB receptor with Co-crystal ligand (MBL) (green)

**S-Fig 4:** showed the molecular docking study of GA on Pi3kϒ receptor. **(a)** The binding mode of GA (brown) is shown in the Pi3kϒ receptor active site and important residues are highlighted with cyan stick, **(b)** Ligplot of GA is shown in the Pi3kϒ receptor active site, **(c)** The binding mode of Co-crystal ligand (A3W)(brown) is shown in the Pi3kϒreceptor active site and important residues are highlighted with cyan stick, **(d)** Ligplot of Co-crystal ligand (A3w) is shown in the Pi3kϒreceptor active site and **(e)** Superimposition of GA (cyan) at catalytic domain of Pi3kϒreceptor with Co-crystal ligand (A3W) (green).

**S-Fig 5:** showed the molecular docking study of GA on JAK-2 (STAT3) receptor. **(a)** The binding mode of GA (brown) is shown in the tyrosine-protein kinase receptor active site and important residues are highlighted with cyan stick, **(b)** Ligplot of GA is shown in the tyrosine-protein kinase receptor active site, **(c)** The binding mode of Co-crystal ligand (LMM)(brown) is shown in the tyrosine-protein kinase receptor active site and important residues are highlighted with cyan stick, **(d)** Ligplot of Co-crystal ligand (LMM) is shown in the tyrosine-protein kinase receptor active site and **(e)** Superimposition of GA (cyan) at catalytic domain of tyrosine-protein kinase receptor with Co-crystal ligand (LMM) (green).

**S-Fig 6:**  The ME regions in Pseudo-ternary phase diagram saw an increase in presence of ethanol, from **A)** 1:1 to **B)** 2:1 ratio.

**S-Fig 7:** Characterization of optimized GA-NLCs: (**7A-B**) shows particle size distribution curve **C)** TEM analysis, and **D)** FE-SEM

**S-Fig 8:** Shows zeta potential of characterized GA-NLC

**S-Fig 9:** *In vitro* release of nano-lipid carrier of ganoderic acid (NLC-GA) and GA solution at different time intervals (0–24 h) using the dialysis method in simulated intestinal fluid of pH 6.8.

**S-Fig 10:** Assessment of cell viability of GA solution, GA-NLC and Blank NLC formulation at different concentration following incubation with HepG2 cells **4A**) for 24 h and **4B**) represents for 48 h duration.

**S-Fig 11:** Illustrated the body effect of GA and GA-NLC treatment on the DEN induced HCC rats. **(a)** Showed the body weight of different group of rats, **(b)** demonstrated the liver tissue weight of different group of rats and **(c)** explained the relative body weight of different group of rats.

**S-Fig 12:** Demonstrated the hepatic parameters effect of GA and GA-NLC treatment on the DEN induced HCC rats. **(a)** AFP, **(b)** CEA, **(c)** GGT, **(d)** AST, **(e)** ALP and **(f)** ALT.

**S-Fig13:** Demonstrated the effect on the non-hepatic parameters of GA and GA-NLC treatment on the DEN induced HCC rats. **(a)** Total protein **(b)** albumin, **(c)** globulin and **(d)** A/G.

**S-Figure 14:** Showing the effect on the antioxidant parameters of GA and GA-NLC treatment on the DEN induced HCC rats. **(a)** GSH, **(b)** MPO, **(c)** GST and **(d)** GPx.

**S-Fig 15:** Demonstrated the effect on the antioxidant parameters of GA and GA-NLC treatment on the DEN induced HCC rats. **(a)** P. carbonyl, **(b)** CAT, **(c)** SOD and **(d)** MDA.

**S-Fig 16:** Shows the effect on the non-antioxidant parameters of GA and GA-NLC treatment on the DEN induced HCC rats **(a)** Vitamin C and **(b)** Vitamin E.
