# Supplementary figures and images for: Ganoderic acid loaded nano-lipidic carriers improvise treatment of hepatocellular carcinoma
Source: Drug Deliv. 2019 Jul 30;26(1):782–93. doi: 10.1080/10717544.2019.1606865 (PMC6711158; doi:10.1080/10717544.2019.1606865)

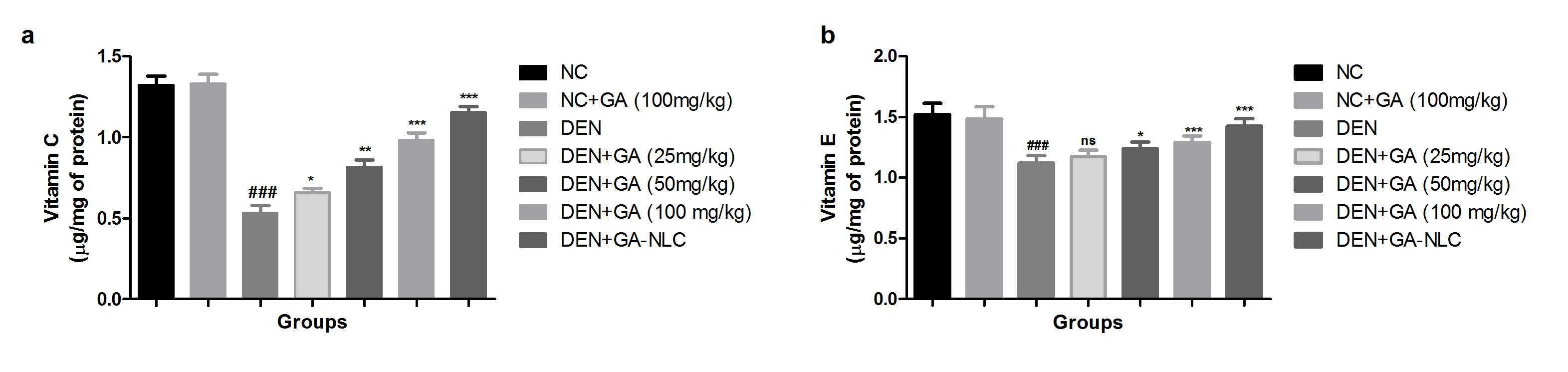

Supplement: S-Fig_16.jpg [file IDRD_A_1606865_SM2296.jpg]

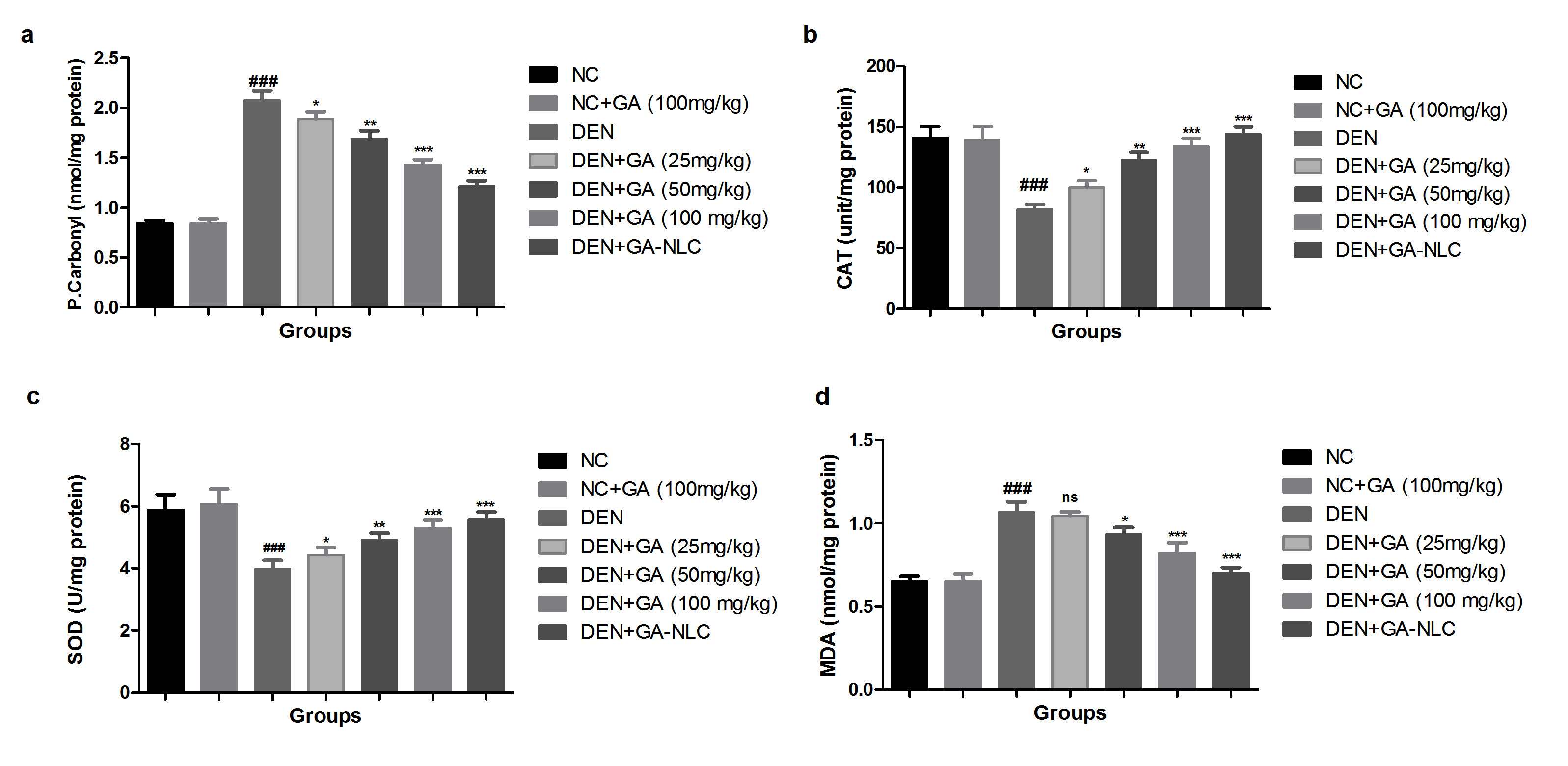

Supplement: S-Fig_15.jpg [file IDRD_A_1606865_SM2295.jpg]

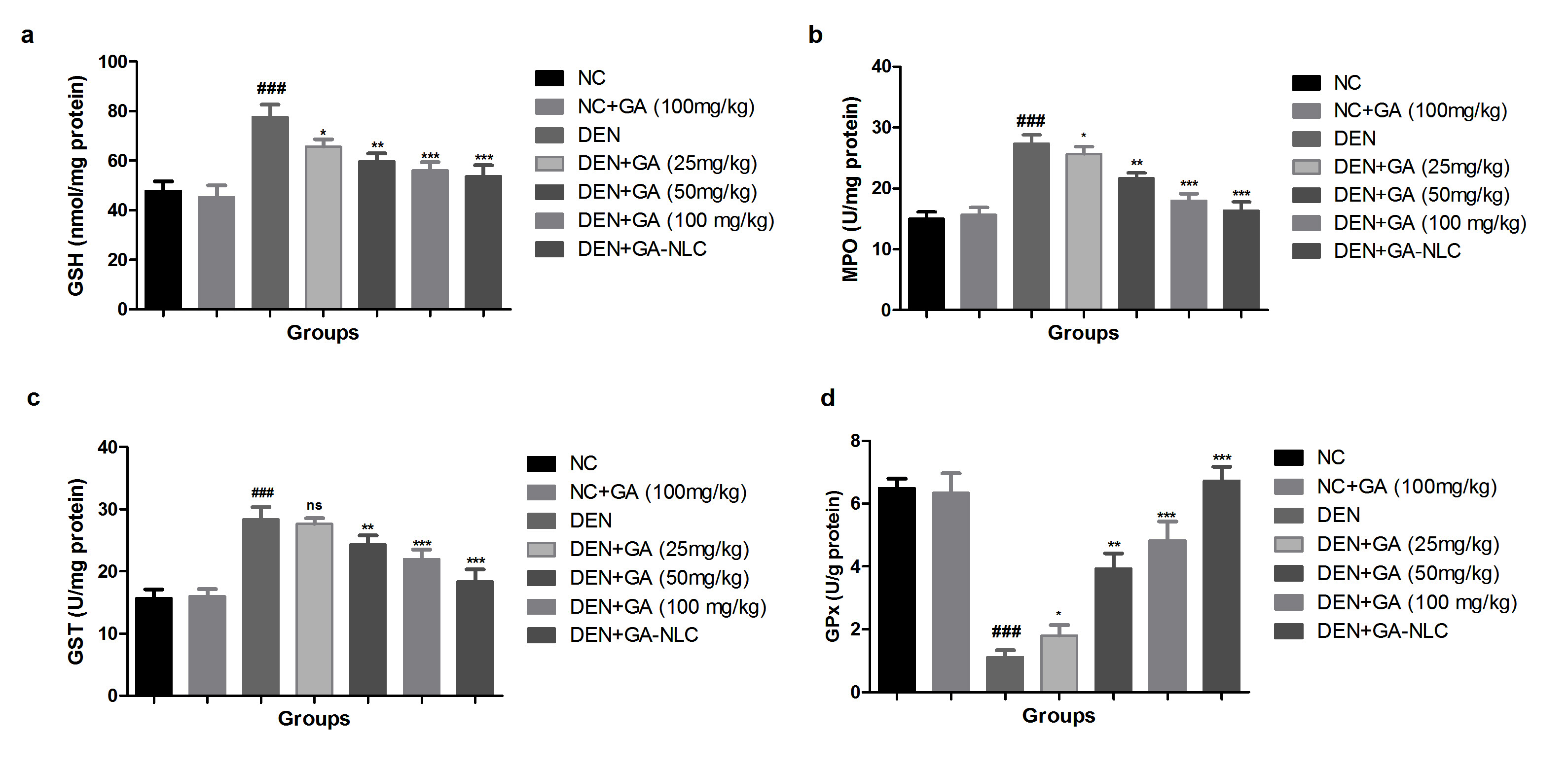

Supplement: S-Fig_14.jpg [file IDRD_A_1606865_SM2294.jpg]

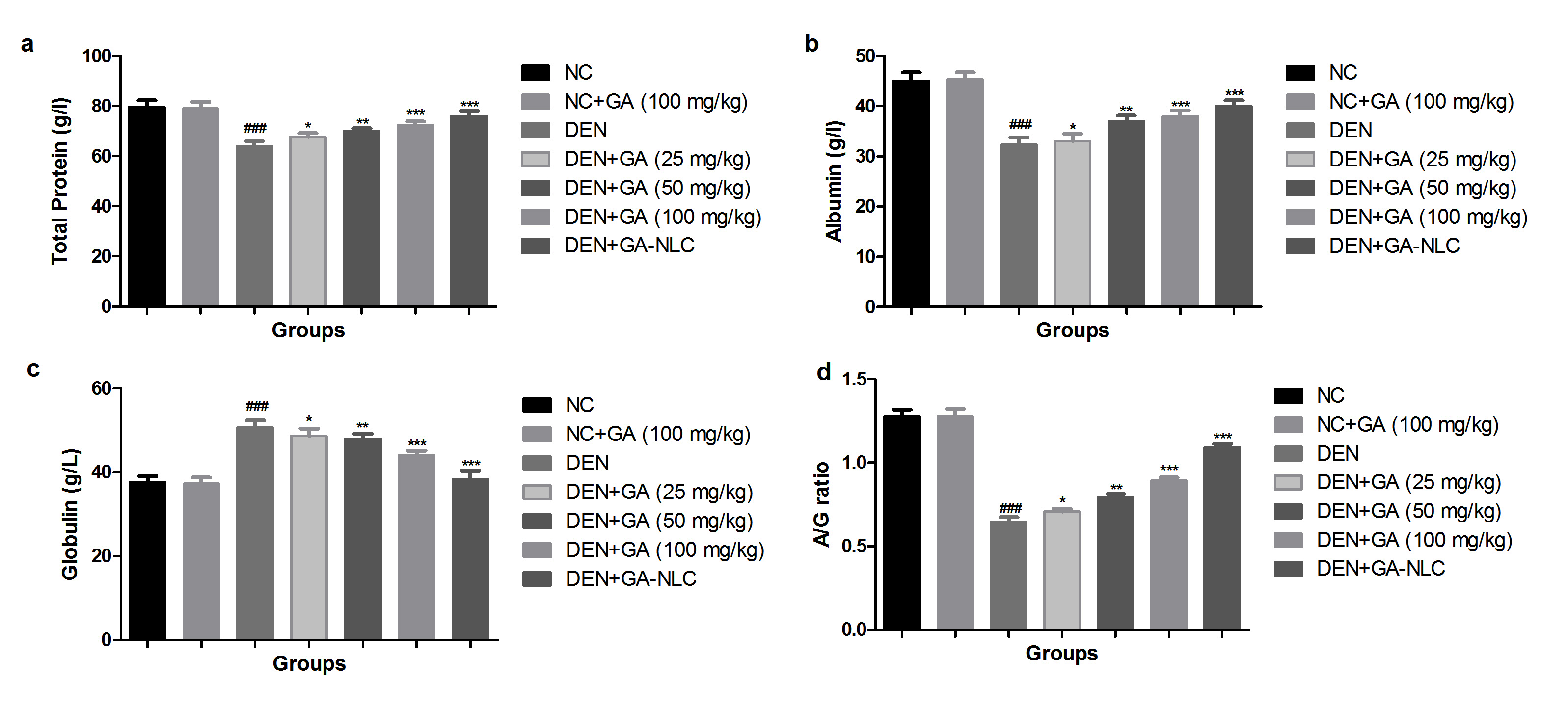

Supplement: S-Fig_13.jpg [file IDRD_A_1606865_SM2293.jpg]

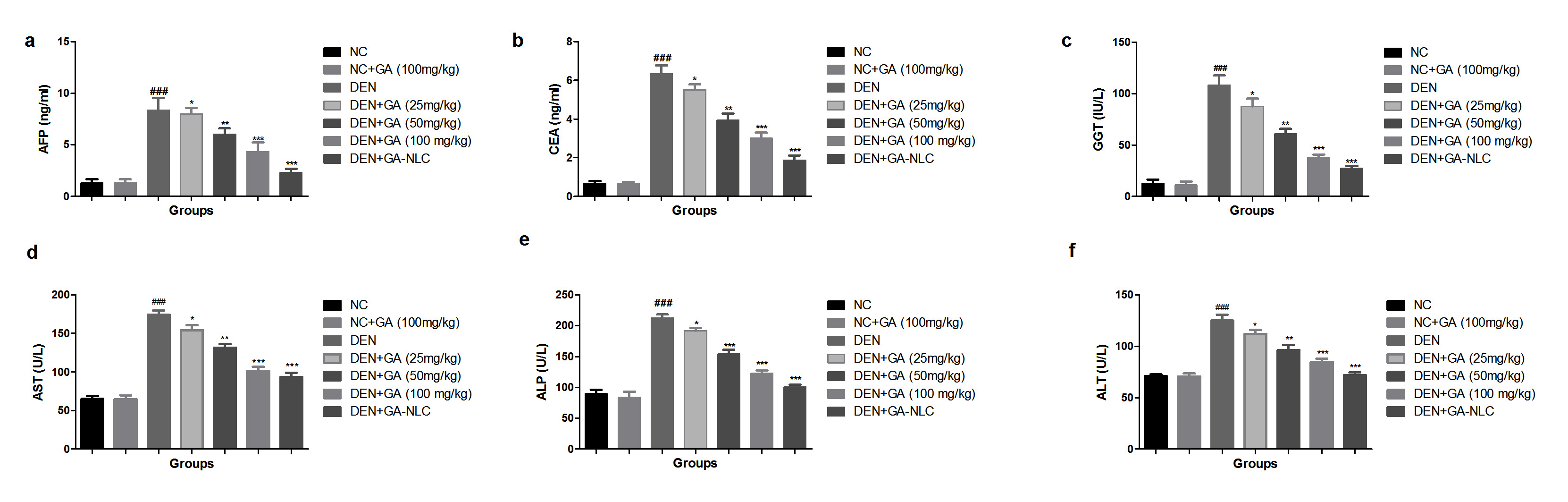

Supplement: S-Fig_12.jpg [file IDRD_A_1606865_SM2292.jpg]

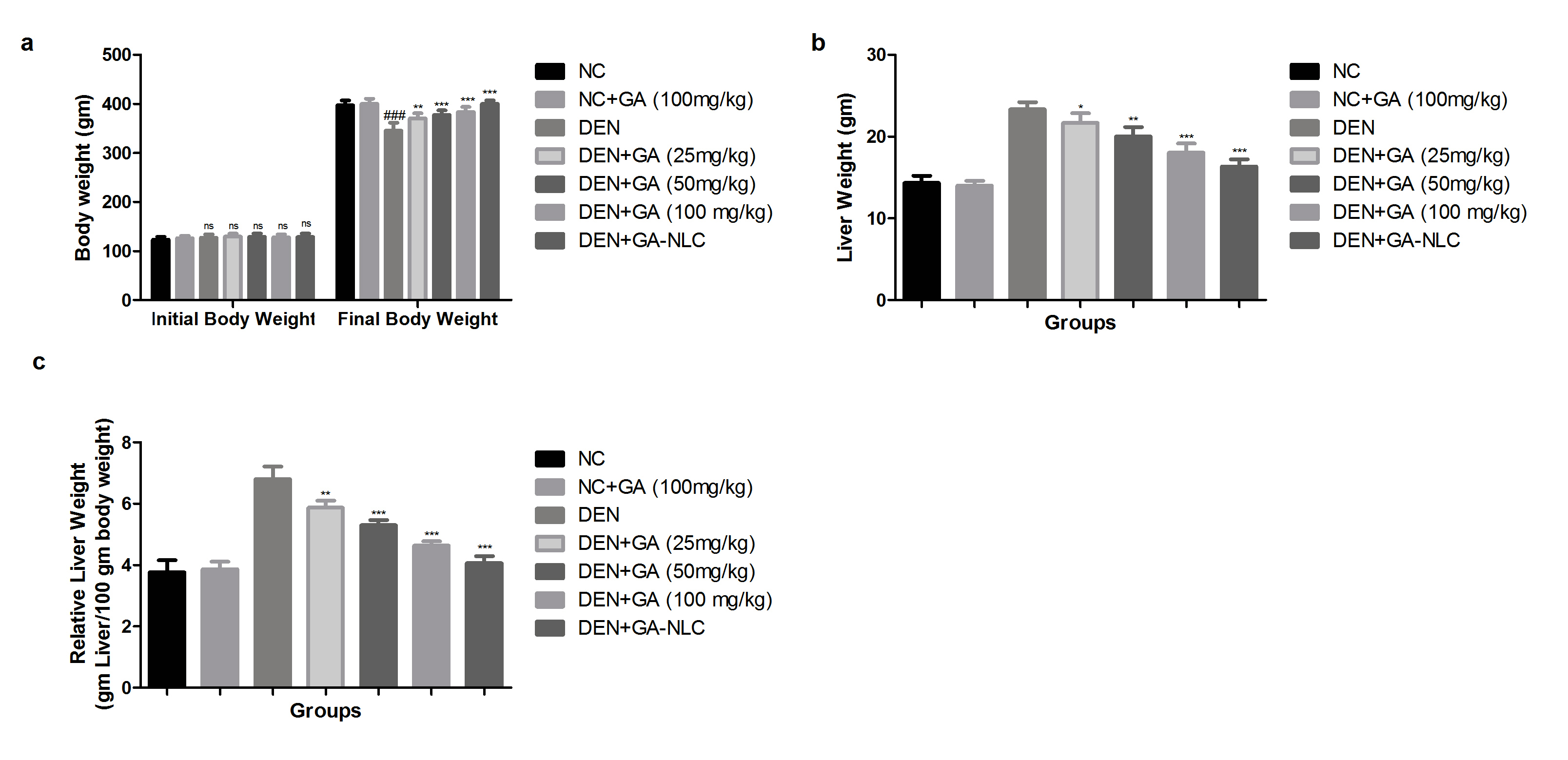

Supplement: S-Fig_11.jpg [file IDRD_A_1606865_SM2291.jpg]

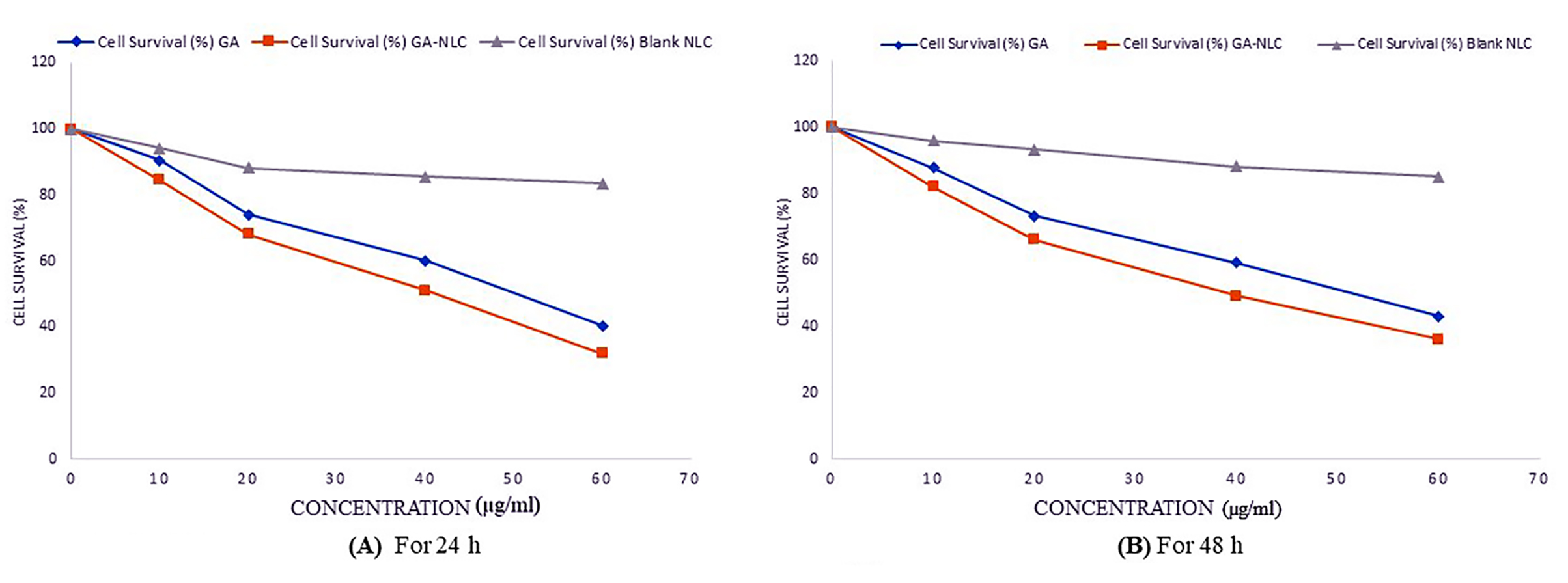

Supplement: S-Fig_10A_and_10B.jpg [file IDRD_A_1606865_SM2290.jpg]

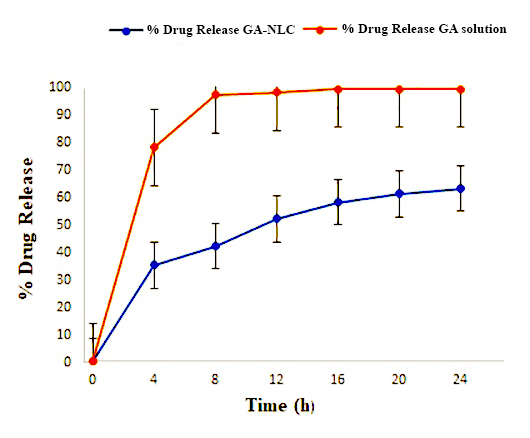

Supplement: S-Fig_9.jpg [file IDRD_A_1606865_SM2289.jpg]

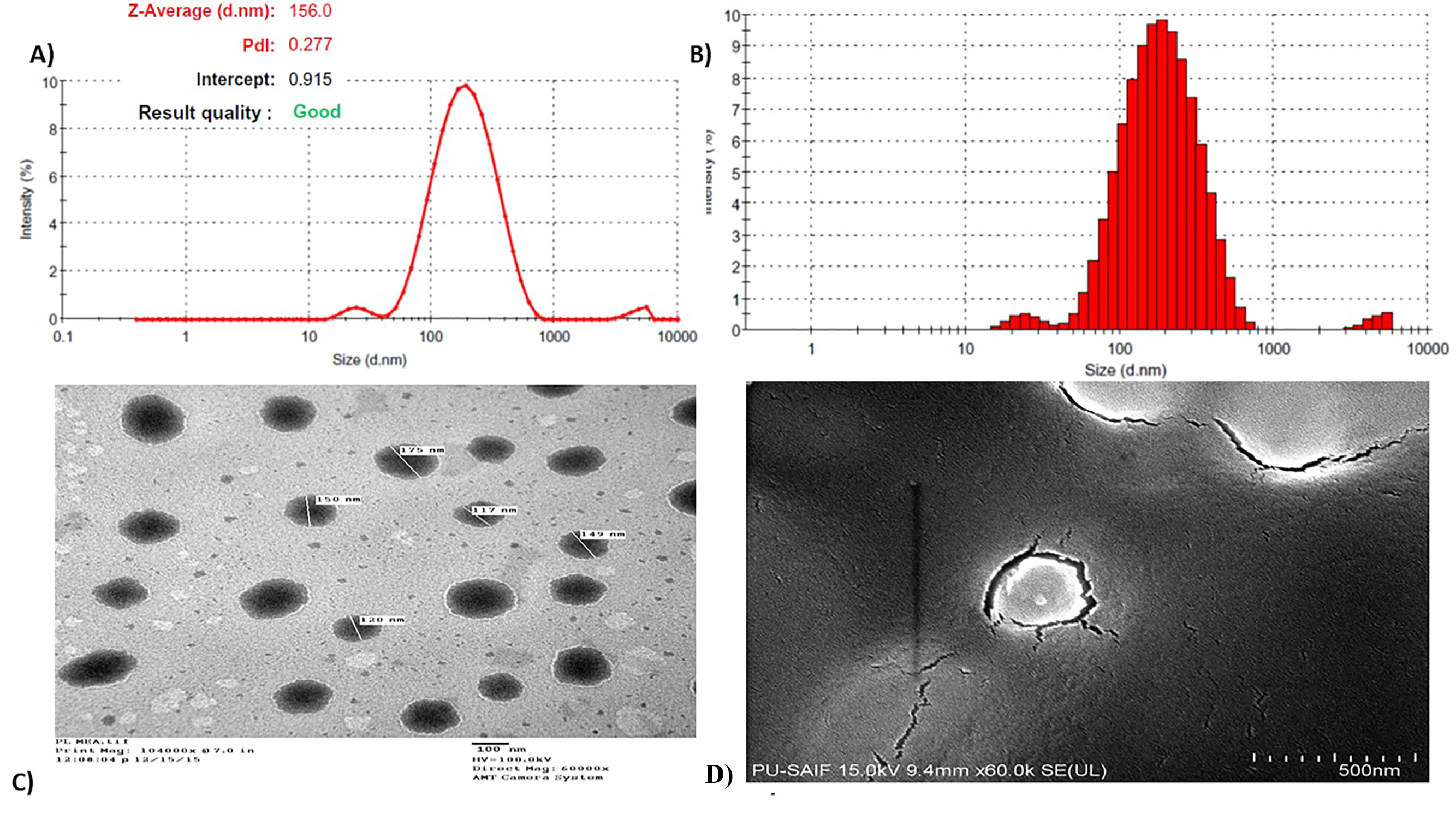

Supplement: S-Fig_7A-D.jpg [file IDRD_A_1606865_SM2287.jpg]

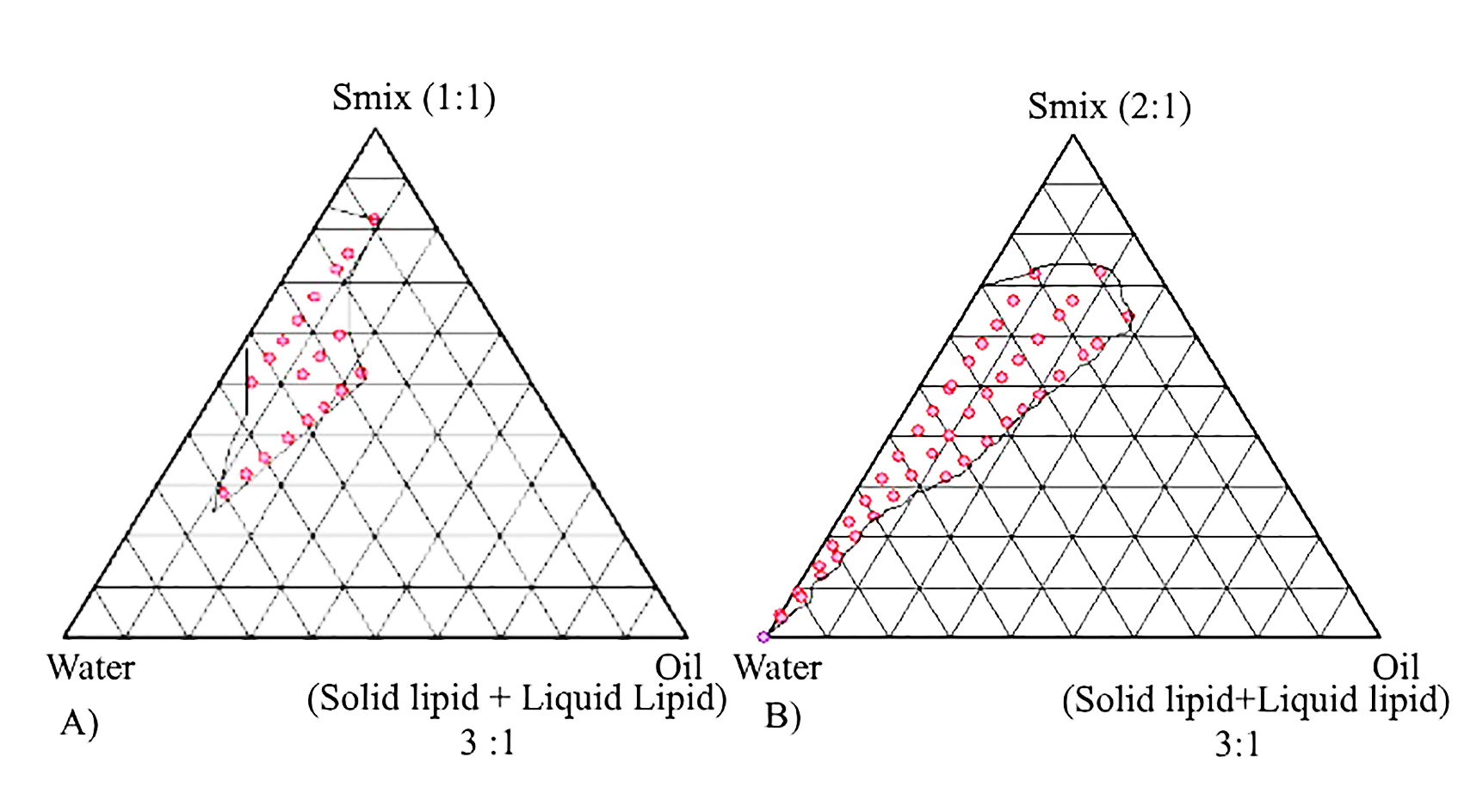

Supplement: S-Fig_6A_and_6B.jpg [file IDRD_A_1606865_SM2286.jpg]

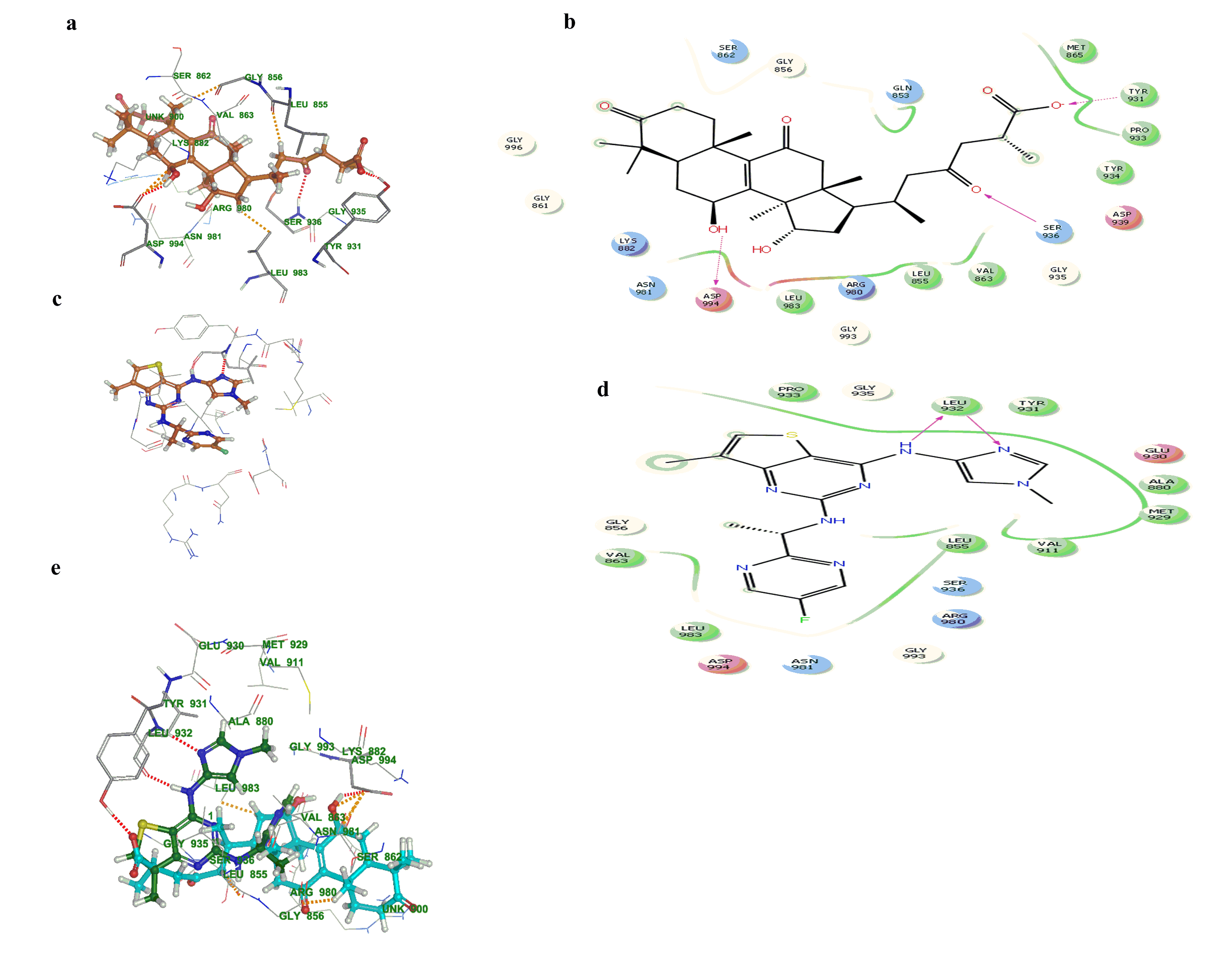

Supplement: S5.tif [file IDRD_A_1606865_SM2285.tif]

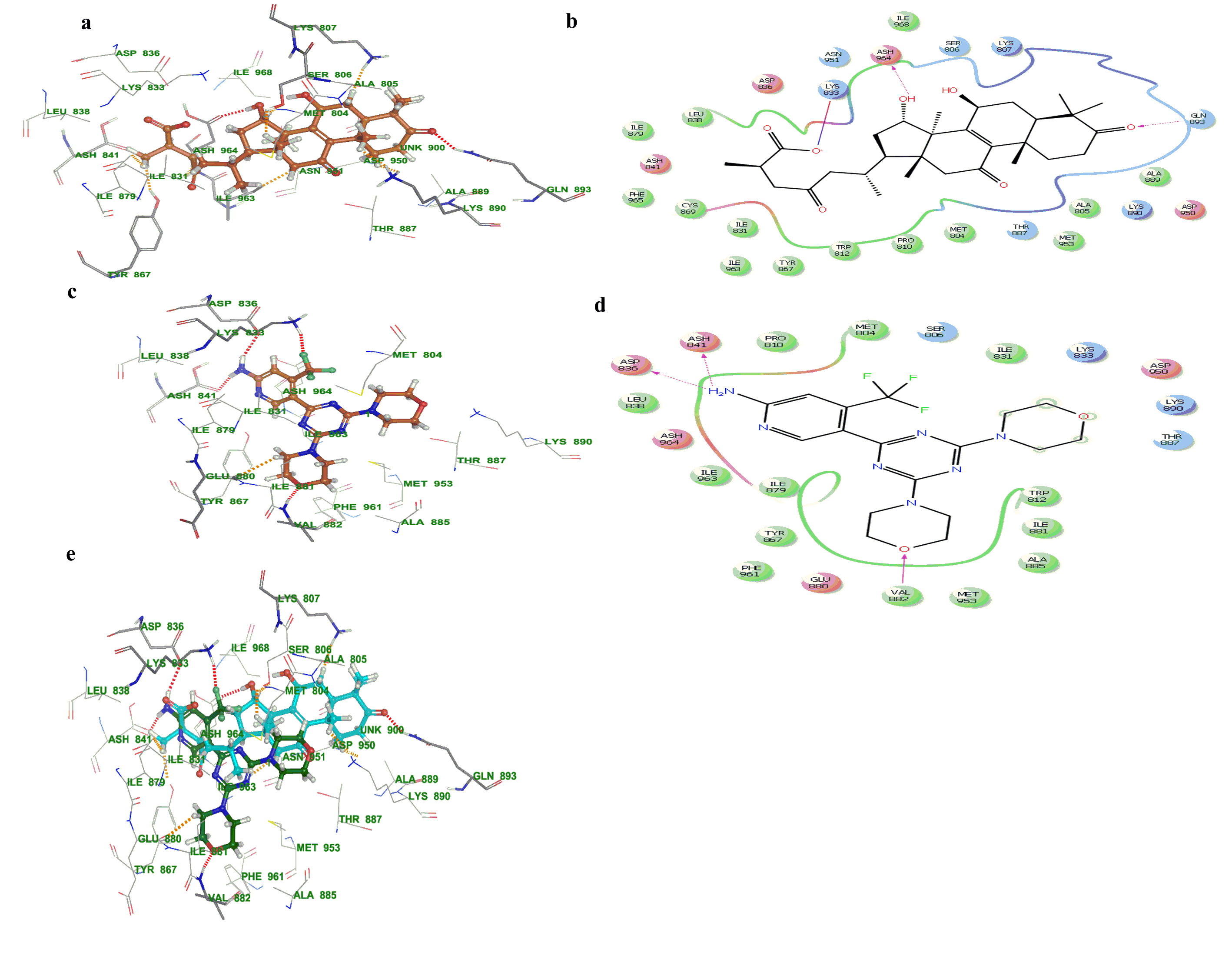

Supplement: S4.tif [file IDRD_A_1606865_SM2284.tif]

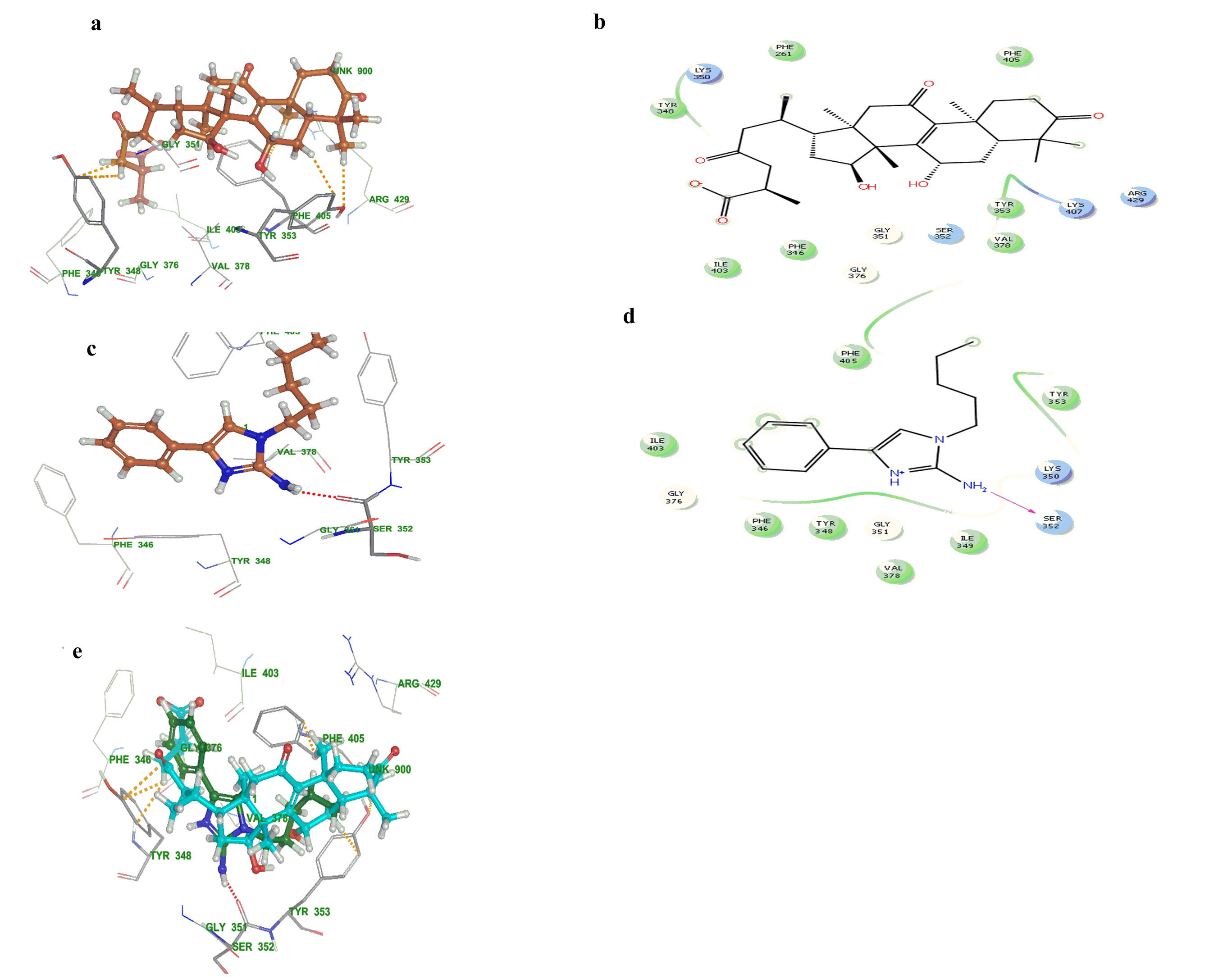

Supplement: S3.tif [file IDRD_A_1606865_SM2283.tif]

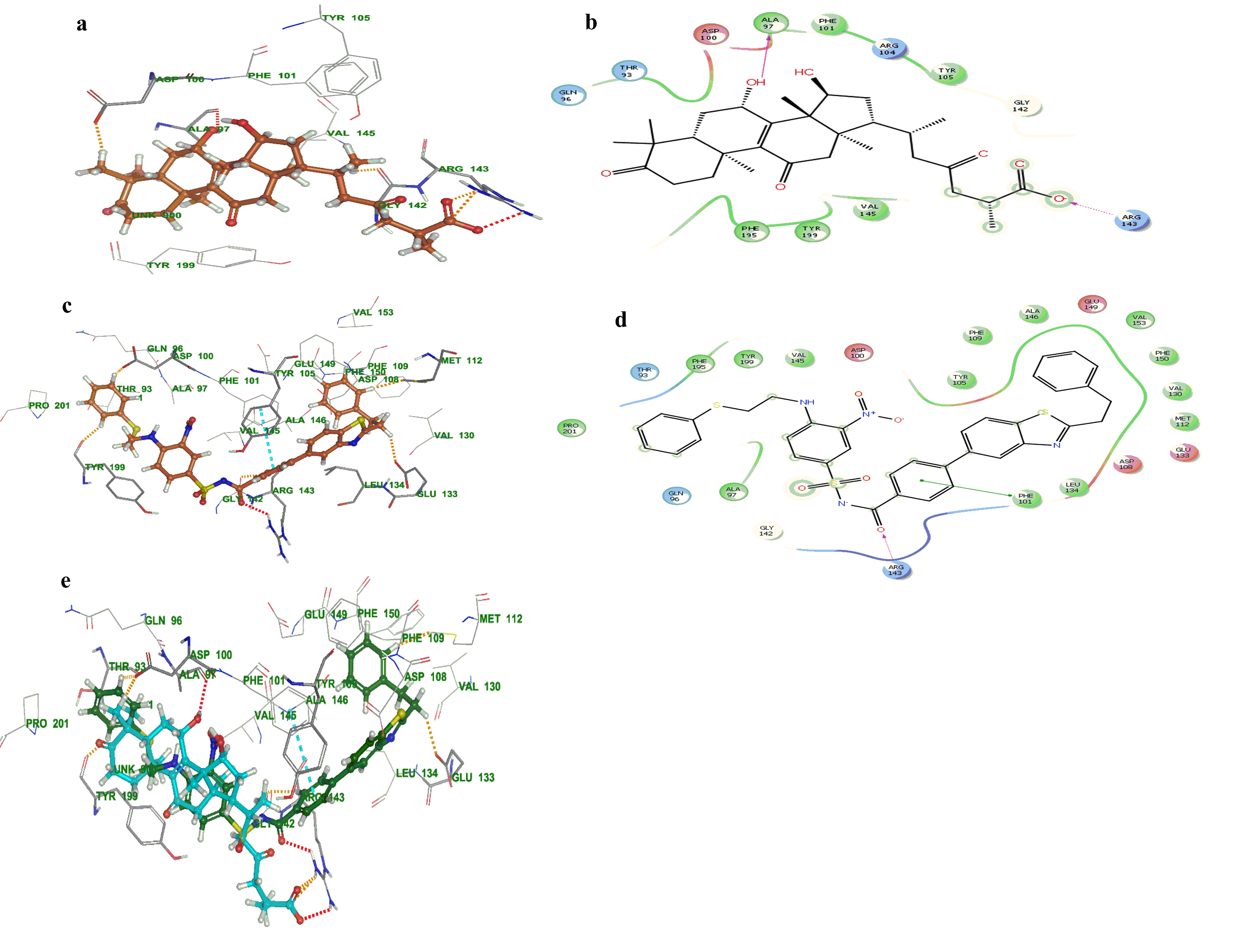

Supplement: S2.tif [file IDRD_A_1606865_SM2282.tif]

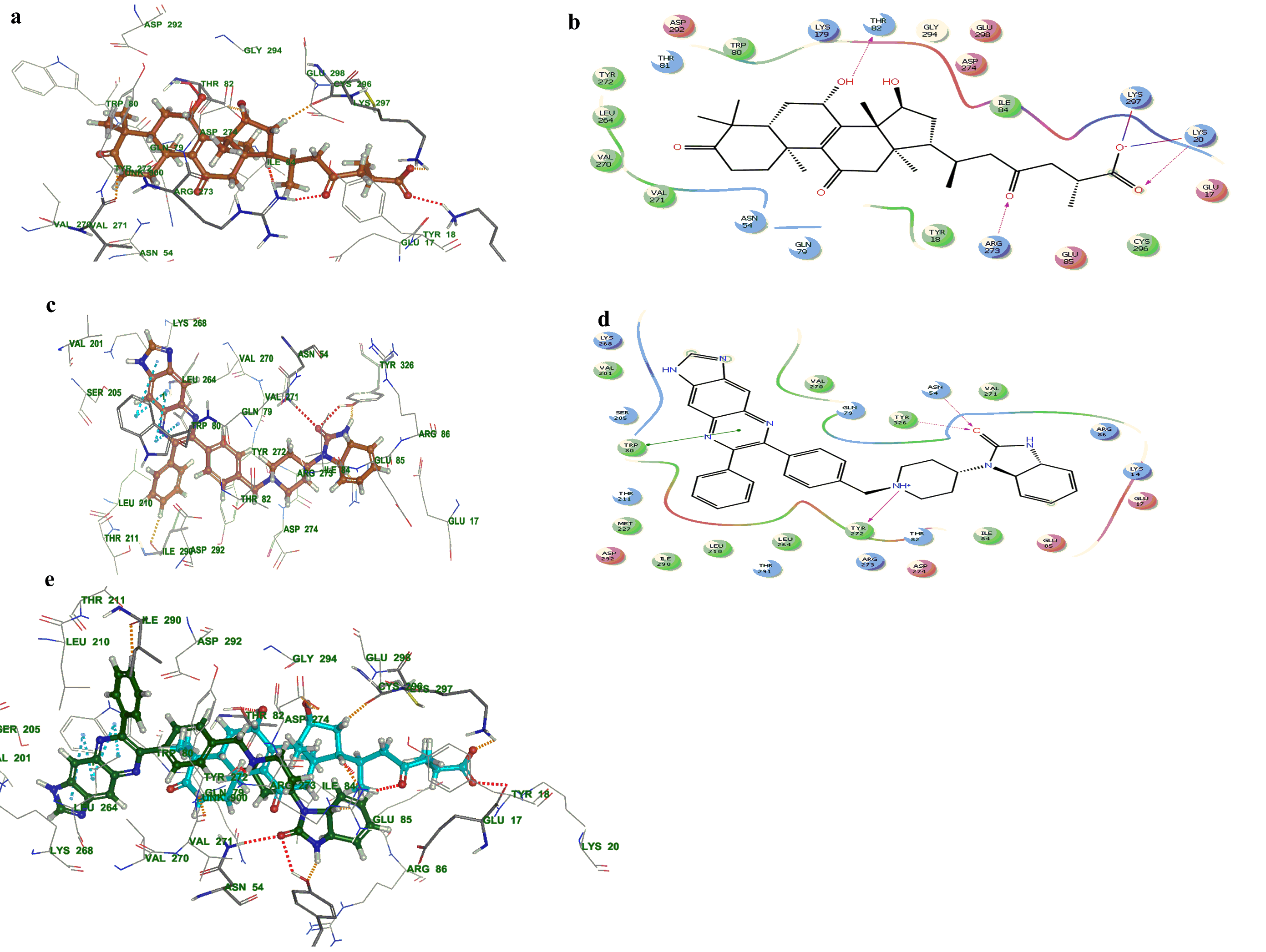

Supplement: S1.tif [file IDRD_A_1606865_SM2281.tif]

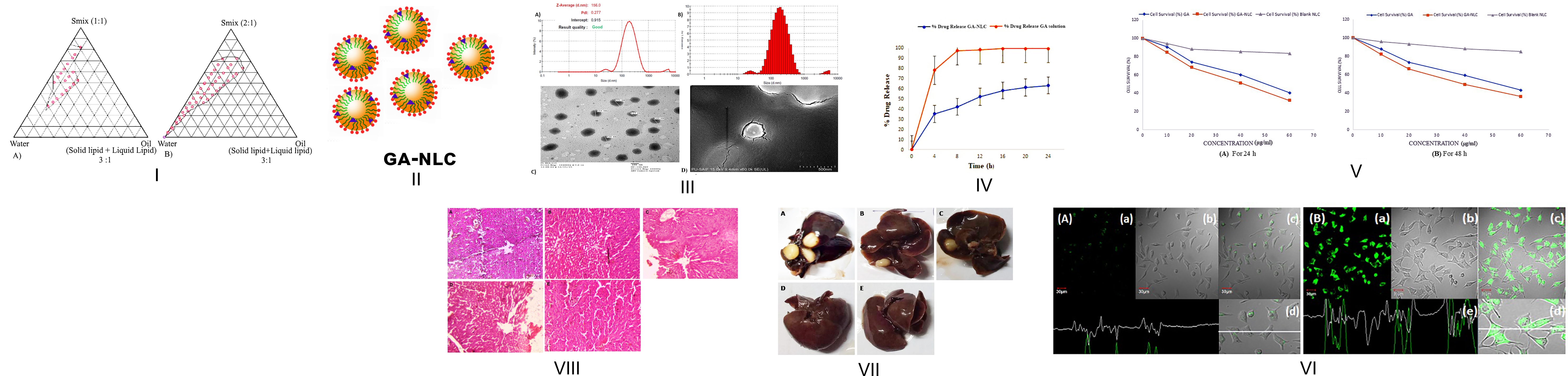

Supplement: Graphical_Abst.jpg [file IDRD_A_1606865_SM2280.jpg]

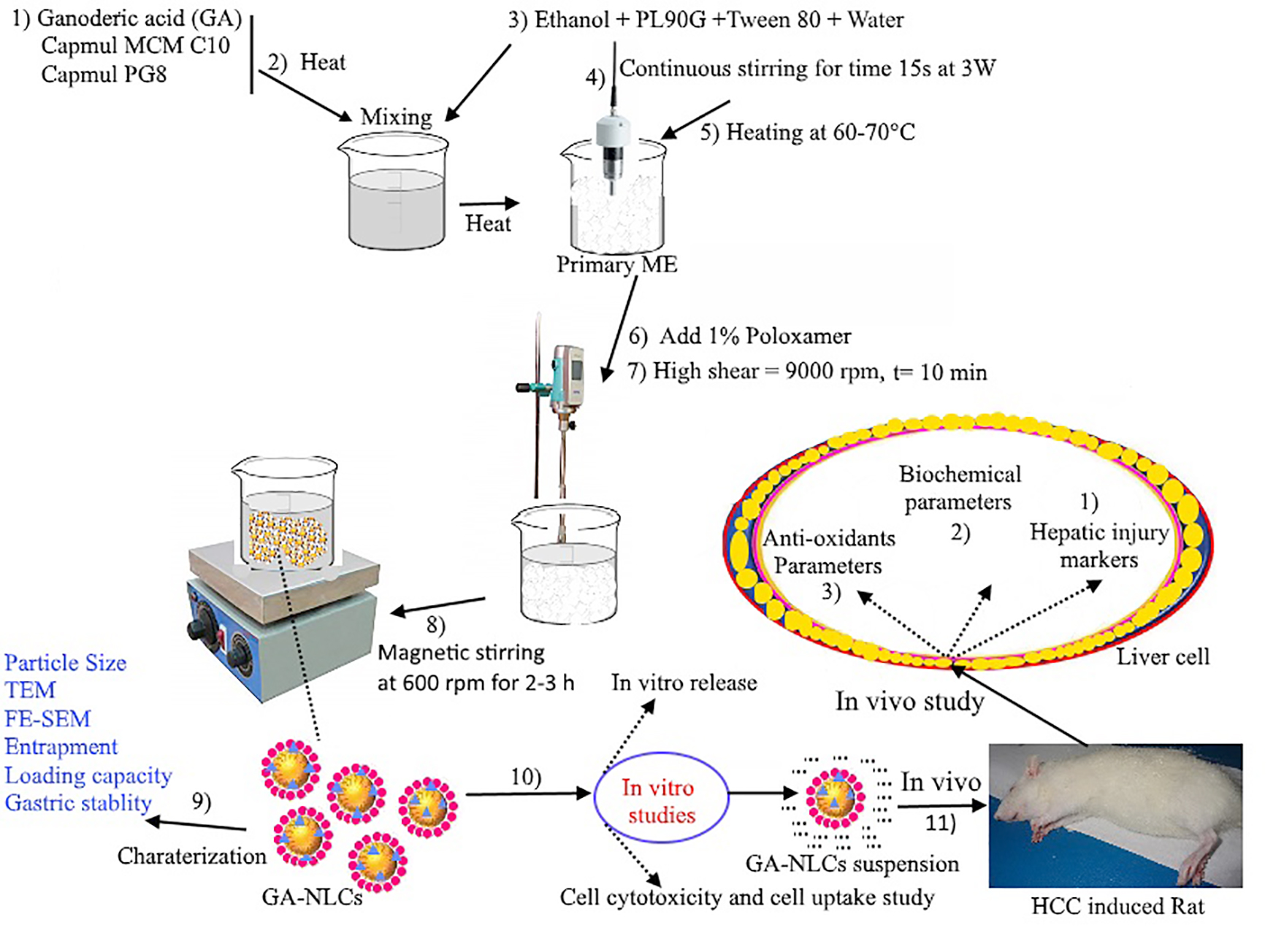

Supplement: S-Scheme_1_corrected.jpg [file IDRD_A_1606865_SM2279.jpg]
