# Supplementary material for: Ganoderic acid loaded nano-lipidic carriers improvise treatment of hepatocellular carcinoma
Source: Drug Deliv. 2019 Jul 30;26(1):782–93. doi: 10.1080/10717544.2019.1606865 (PMC6711158; doi:10.1080/10717544.2019.1606865)

# Zeta Potential Report

*v2.3*

Malvern Instruments Ltd - © Copyright 2008

## Sample Details

**Sample Name:** C 1

**SOP Name:** mansettings.nano

**General Notes:**

| | | | |
|---|---|---|---|
| **File Name:** | Example Result3.dts | **Dispersant Name:** | Water |
| **Record Number:** | 46 | **Dispersant RI:** | 1.330 |
| **Date and Time:** | Monday, November 23, 2015 4:57... | **Viscosity (cP):** | 0.8872 |
| | | **Dispersant Dielectric Constant:** | 78.5 |

## System

| | | | |
|---|---|---|---|
| **Temperature (°C):** | 25.0 | **Zeta Runs:** | 18 |
| **Count Rate (kcps):** | 543.5 | **Measurement Position (mm):** | 2.00 |
| **Cell Description:** | Clear disposable zeta cell | **Attenuator:** | 6 |

## Results

| | | | Mean (mV) | Area (%) | St Dev (mV) |
|---|---|---|---|---|---|
| **Zeta Potential (mV):** | -4.99 | **Peak 1:** | 0.00 | 0.0 | 0.00 |
| **Zeta Deviation (mV):** | 0.00 | **Peak 2:** | 0.00 | 0.0 | 0.00 |
| **Conductivity (mS/cm):** | 15.3 | **Peak 3:** | 0.00 | 0.0 | 0.00 |

**Result quality :** **See result quality report**

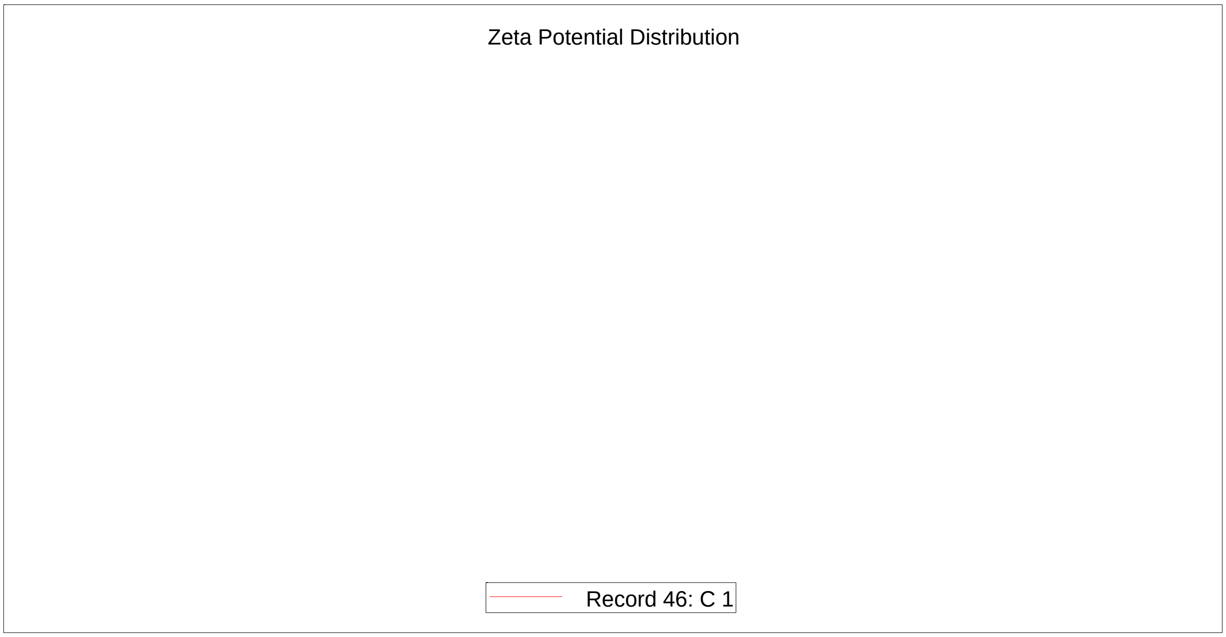

Supplement: S_Fig-8.pdf [file IDRD_A_1606865_SM2288.pdf]
