# Supplementary material for: Ganoderic acid loaded nano-lipidic carriers improvise treatment of hepatocellular carcinoma
Source: Drug Deliv. 2019 Jul 30;26(1):782–93. doi: 10.1080/10717544.2019.1606865 (PMC6711158; doi:10.1080/10717544.2019.1606865)
Supplement: Supplementary_Tables.docx [file IDRD_A_1606865_SM2278.docx]

**Supplementary Table 1:** Shows Ganoderic acid binding interaction with backbone of RAC-alpha serine/threonine-protein kinase receptor, Bcl-2 receptor, NF-kB receptor, Pi3kϒ receptor, and JAK-2 (STAT3) receptors.

| Target | PDB ID | Hydrogen Bonds | Hydrophobic interactions | Dock/Grid score | Binding energy  for Ganoderic acid (kcal/Mol) | -log(Kd) for Ganoderic acid | Binding energy for  known inhibitor  (Kcal/Mol) | -log(Kd) for known  inhibitor |
| --- | --- | --- | --- | --- | --- | --- | --- | --- |
| RAC-alpha serine/threonine-protein kinase | 3o96 | 03 | 04 | -9.43 | -8.31 | 4.53 | -9.85 | 7.55 |
| Apoptosis regulator Bcl-2 | 2o21 | 02 | 02 | -3.93 | -7.89 | 5.28 | -8.65 | 8.49 |
| **NF-kB** | 5az5 | 00 | 03 | -4.11 | -8.89 | 6.56 | -9.12 | 7.12 |
| **Pi3kϒ** | 5oq4 | 03 | 06 | -3.39 | -5.63 | 7.89 | -6.52 | 9.85 |
| **STAT3** | 4c61 | 03 | 04 | -5.11 | -10.65 | 5.36 | -11.23 | 5.65 |

**Supplementary Table 2**: ADMET analysis and MM/GBSA Assay data

| **S.N.** | **Compound Code** | **PDBID** | **QP log Po/w^a^** | **HOA^b^** | **Rule of Five^c^** | **∆G_bind_** |
| --- | --- | --- | --- | --- | --- | --- |
| 1 | **Ganoderic Acid** | 3o96 | 4.53 | 2 | 1 | -84.834 |
| 2 | **Ganoderic Acid** | 2o21 | NA | NA | NA | -41.02 |
| 3 | **Ganoderic Acid** | 5az5 | NA | NA | NA | -40.09 |
| 4 | **Ganoderic Acid** | 5oq4 | NA | NA | NA | -15.37 |
| 5 | **Ganoderic Acid** | 4c61 | NA | NA | NA | -69.21 |
| 6 | IQO | 3o96 | 5.78 | 1 | 2 | -95.67 |
| 7 | 43B | 2o21 | 7.55 | 1 | 2 | -38.52 |
| 8 | MBL | 5az5 | 3.54 | 3 | 0 | -42.65 |
| 9 | A3W | 5oq4 | 2.41 | 3 | 0 | -16.59 |
| 10 | LMM | 4c61 | 3.76 | 3 | 0 | -32.53 |

^a^Predicted octanol/water partition coefficient (<5).

^c^Lipinski’s violations (≤1).

^b^Human Oral Absorption 1, 2 and 3 for low, medium and high respectively

**Supplementary Table 3.** Characterization of optimized GA-NLCs formulations. Data are shown as mean ±SD. (n=6).

| **Formulation** | **Particle size (nm)** | **PDI** | **Zeta potential (mV)** | **Drug loading capacity** | **EE (%)** |
| --- | --- | --- | --- | --- | --- |
| GA-NLCs | 156 ± 0.5 nm | 0.277 | -4.99 **±** 1.3 mV | 12.2 ± 2.11 | 86.3 ±1.5 |

| **Stability Parameters** | **SGF (pH 1.2) SIF (pH 6.8)** |
| --- | --- |
|  | **Before After Before After** |
| **Particle size (nm)** | 156 ± 0.5 nm 158 ± 1.20 nm 157 ± 1.05 nm 157.8 ± 1.24 nm |
| **PDI** | 0.277 0.31 0.288 0.33 |
| **Zeta potential** | -4.99 ± 1.3 mV -5.1± 0. 11 mV -3.45 ± 0.12 mV -3.92 ± 0.22 mV |
| **Entrapment Efficiency (%)** | 86.3±1.5 85.12± 0.12 87.12± 1.31 85.22± 1.11 |

**Supplementary Table 4:** *In vitro* Gastrointestinal stability of characterized NLC in different dissolution media.

SGF: Simulated gastric fluid; SIF: Simulated intestinal fluid; PDI: Polydispersity Index

**Supplementary Table 5:** GA-NLC, its effects on number of rats with tumour incidence.

| **S. No** | **Groups** | **Number of rats/Number of rats with tumour** | **Tumour incidence (%)** |
| --- | --- | --- | --- |
| **1** | **DEN Control** | 9/9 | 100 |
| **2** | **DEN+GA (25 mg/kg)** | 10/11 | 90.90 |
| **3** | **DEN+GA (50 mg/kg)** | 7/10 | 70 |
| **4** | **DEN+ GA (100 mg/kg)** | 4/9 | 44.44 |
| **5** | **DEN+ GA-NLC** | 2/9 | 22.23 |

Group I and group II did not show the any sign of hepatic nodules
